# Growth parameters of *Liberibacter crescens* suggest ammonium and phosphate as essential molecules in the *Liberibacter*-plant host interface

**DOI:** 10.1186/s12866-019-1599-z

**Published:** 2019-10-12

**Authors:** Maritsa Cruz-Munoz, Alam Munoz-Beristain, Joseph R. Petrone, Matthew A. Robinson, Eric W. Triplett

**Affiliations:** 1 Microbiology and Cell Science Department, Institute of Food and Agricultural Sciences, University of Florida, Gainesville, FL USA; 2 Biostatistics Department, College of Health Professions and College of Medicine, University of Florida, Gainesville, FL USA

**Keywords:** *Liberibacter*, Lifestyle, Growth parameters, pH, Aeration, Temperature, Buffering capacity, Ammonium, Phosphate, Concentrations, Pathogens, Disease, Management

## Abstract

**Background:**

*Liberibacter crescens* is the closest cultured relative of four important uncultured crop pathogens. *Candidatus. L. asiaticus*, *L. americanus*, *L. africanus* cause citrus greening disease, while Ca. L. solanacearum causes potato Zebra chip disease. None of the pathogens grows in axenic culture. *L. crescens* grows in three media: a BM-7, a serum-free Hi® Grace’s Insect Medium (Hi-GI), and a chemically-defined medium called M15. To date, no optimal growth parameters of the model species *L. crescens* have been reported. Studying the main growth parameters of *L. crescens* in axenic culture will give us insights into the lifestyle of the Ca. *Liberibacter* pathogens.

**Results:**

The evaluation of the growth parameters—pH, aeration, temperature, and buffering capacity—reflects the optimal living conditions of *L. crescens.* These variables revealed that *L. crescens* is an aerobic, neutrophilic bacterium, that grows optimally in broth in a pH range of 5.8 to 6.8, in a fully oxygenated environment (250 rpm), at 28 °C, and with monosodium phosphate (10 mM or 11.69 mM) as the preferred buffer for growth. The increase of pH in the external media likely results from the deamination activity within the cell, with the concomitant over-production of ammonium in the external medium.

**Conclusion:**

*L. crescens* and the Ca. Liberibacter pathogens are metabolically similar and grow in similar environments—the phloem and the gut of their insect vectors. The evaluation of the growth parameters of *L. crescens* reveals the lifestyle of *Liberibacter*, elucidating ammonium and phosphate as essential molecules for colonization within the hosts. Ammonium is the main driver of pH modulation by active deamination of amino acids in the *L. crescens* amino acid rich media. In plants, excess ammonium induces ionic imbalances, oxidative stress, and pH disturbances across cell membranes, causing stunted root and shoot growth and chlorosis—the common symptoms of HLB-disease. Phosphate, which is also present in Ca. *L. asiaticus* hosts, is the preferred buffer for the growth of *L. crescens*. The interplay between ammonium, sucrose, potassium (K^+^), phosphate, nitrate (NO_3_^−^), light and other photosynthates might lead to develop better strategies for disease management.

## Background

The genus *Liberibacter* comprises reduced-genome, fastidious α-proteobacteria including the not yet cultured Ca. Liberibacter species, which are responsible for the devastating citrus greening, or Huanglongbing (HLB), and potato zebra chip (ZC) diseases worldwide [1]. Three species—Ca. *L. asiaticus*, Ca. *L. americanus*, and Ca. *L. africanus*—are the casual agents of HLB while Ca. L. solanacearum is responsible for potato ZC [2]. In Florida, citrus production has been severely affected since the discovery the citrus greening disease in 2005 [3]. The pathogenic Ca. Liberibacter species are transmitted into the phloem of citrus and potato plants by phloem feeders from the family psyllidae [2]. The genus *Liberibacter* also includes the endophytic bacterium Ca. *L. europaeus* found in carrots and celery as well as the only culturable species, *L. crescens*, which was isolated from mountain papaya in 1995 [4, 5]. *L. crescens* might be a non-pathogenic species because several attempts to inoculate it into citrus, mountain papaya, dodder, and the Asian citus psyllid—*Diaphorina citri,* have all failed [6]. Compared to Ca. Liberibacter pathogens, *L. crescens* contains a slightly larger genome, which most likely confers the metabolic capabilities to grow in vitro [7, 8]. Since *L. crescens* is the sole cultured member of the genus *Liberibacter*, it is the best and closest model bacteria to study the lifestyle and metabolism of Ca. Liberibacter pathogens.

Fast-growing model bacteria such as *Escherichia coli* and *Bacillus subtilis* grow in complex and chemically-defined media in which the optimal chemical and physical growth parameters have been determined [9]. In the genus *Liberibacter,* the only species in which these conditions can be studied is *L. crescens.* Chemical factors such as carbon sources and essential nutrients were recently studied for *L. crescens* in the M15 chemically-defined medium [10]. Here, citrate was proposed as the preferred carbon source for the growth of *L. crescens* at slightly acid conditions. Citrate is abundant in the citrus phloem and present in the psyllid haemolymph where Ca. *L. asiaticus* thrives [11, 12]. Physical factors such as pH, aeration, and temperature [13] have not been fully described for *L. crescens* in axenic culture, except for the optimal temperature for growth at 27–28 °C and an initial pH of 6.5 [4, 8].

Here, the optimal growth conditions of *L. crescens* are presented for four growth parameters—pH, aeration, temperature and buffering capacity—using one factor at a time methodology (OFAT) [14]. The evaluation of the growth parameters of *L. crescens* gave insight into the physiology, lifestyle and culturability of the Ca. Liberibacter pathogens. The growth parameters indicated that *L. crescens* grows optimally in an initial pH of 5.8 to 6.8, in a fully oxygenated environment, at 28 °C, and monosodium phosphate as the most suitable buffer for growth. Similar to *L. crescens,* Ca. Liberibacter asiaticus might also require acidic conditions to grow because the citrus phloem is acidic (5.0–5.74) [11]. Ca. Liberibacter species are also expected to be aerobic pathogens because they contain the complete TCA cycle and respiratory chain machinery [8]. The optimal temperatures in which Ca. *L. asiaticus* grew in the citrus phloem is also at 22/27 °C night/day photoperiod [15]. Phosphoric acid is also present in the citrus phloem (1.14–9.16 mM), which might be an indication of the phosphorus requirements for Ca. *L. asiaticus* growth [11].

The evaluation of the growth parameters of *L. crescens* also highlighted the mechanisms of pH modulation for *Liberibacter* growth*.* In gram-negative neutrophilic aerobic bacteria such as *Liberibacter* species the acid tolerance response (ATR) is activated when they are exposed to slightly acidic conditions. ATR mechanisms include the consumption of protons through decarboxylation of amino acids, the consumption of protons through the respiratory chain, and ammonia generation through active deamination of amino acids [16, 17]. Therefore, the increase of pH in relation to growth in *L. crescens* cultures most likely resulted from the accumulation of ammonium in milimolar concentrations in the external cell culture media. Similarly in the amino acid-rich phloem sap [11, 18], Ca. *L. asiaticus* might be deaminating amino acids with the concomitant over production of ammonium. Excess ammonium is toxic for plants, inducing ionic imbalances, oxidative stress, and disturbances of the pH gradients across plant membranes [19]. These events lead to leaf chlorosis and inhibition of root and shoot growth, which are also some of the common symptoms of HLB-infected trees [19, 20].

Studying the factors that influence the growth of *L. crescens* will contribute to the understanding of the physiology and the lifestyle of the Ca. Liberibacter pathogens. The optimal living conditions, the discovery of phosphate as the preferred buffer for *L. crescens* growth, and the accumulation of ammonium in *L. crescens* cultures open new avenues to understand Ca. Liberibacter culturing and to develop nutritional strategies utilizing sucrose, phosphate, potassium, nitrate, and light to alleviate HLB-symptoms [21–26].

## Results

### Growth parameters of *Liberibacter crescens* in axenic culture

**Fig. 1:**
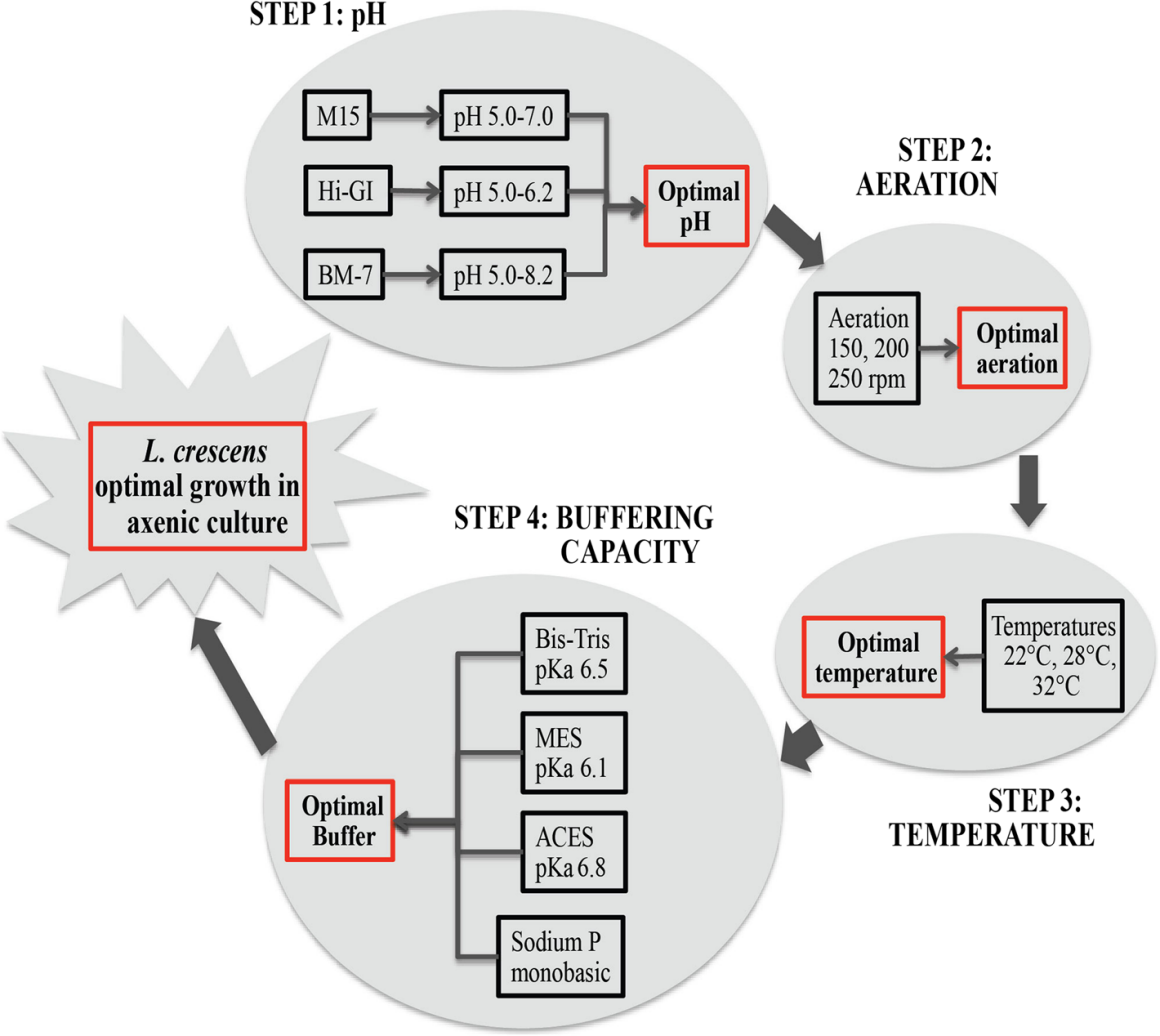
Strategy for the evaluation of the growth parameters for *L. crescens* using OFAT methodology. The evaluation strategy included four variables: pH, aeration, temperature, and buffering capacity of the different growth media—BM-7, Hi-GI, and M15

The OFAT methodology allows studying one variable at a time, and implementing its optimal conditions to the analyses of the subsequent variables [14]. The OFAT methodology to study the growth parameters of *L. crescens* is evaluated using four criteria: pH optimization, aeration, temperature preference, and buffer requirements (Fig. 1)*.* Since phosphate was the preferred buffer for *L. crescens* growth, phosphate concentrations based on the levels found in the citrus phloem were also studied for *L. crescens* growth [11]. The mechanisms of pH modulation are also presented showing that accumulation of ammonium in the M15 chemically-defined medium is the major factor inducing the pH increase in *L. crescens* cultures. Finally, cell viability is also presented for the optimal growth conditions in the three media—M15, Hi-GI and BM-7 media.

### First step: evaluating the optimal pH for *L. crescens* growth

**Fig. 2:**
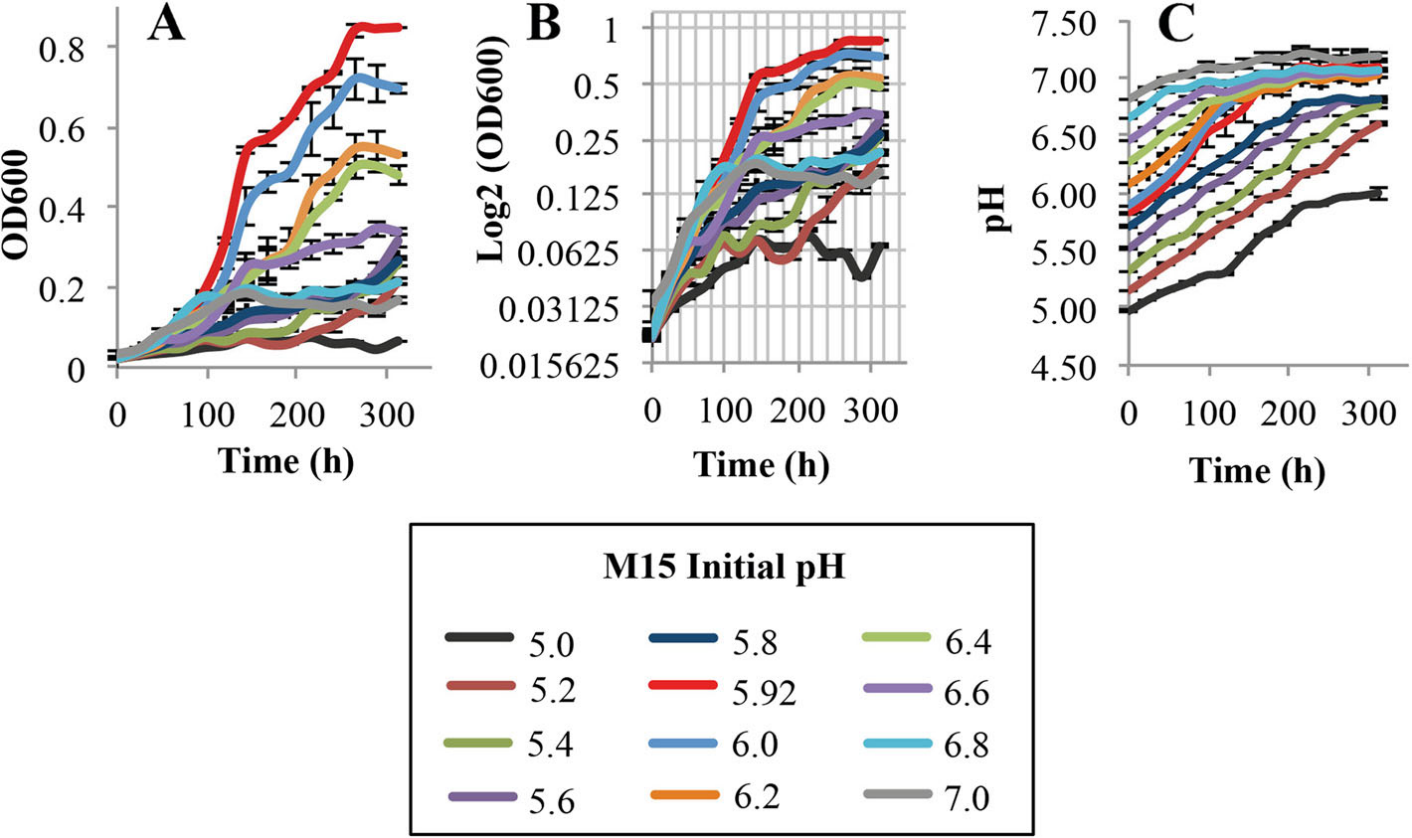
*L. crescens* growth with pH at a range of initial pH (5.0–7.0) in M15 medium. *L. crescens* was grown to exponential phase in Hi-GI at 150 rpm, 28 °C (OD_600_~0.5–0.6, Fig. 15) and washed with a sterile ACES-KOH buffer pH 6.5 prior to inoculation. Optimal growth is visualized at pH 5.92. **a** Cell growth represented as optical density, **b** Cell growth in Logarithmic (log2) scale of the optical density, and **c** pH of the external medium over time during cell growth

**Fig. 3:**
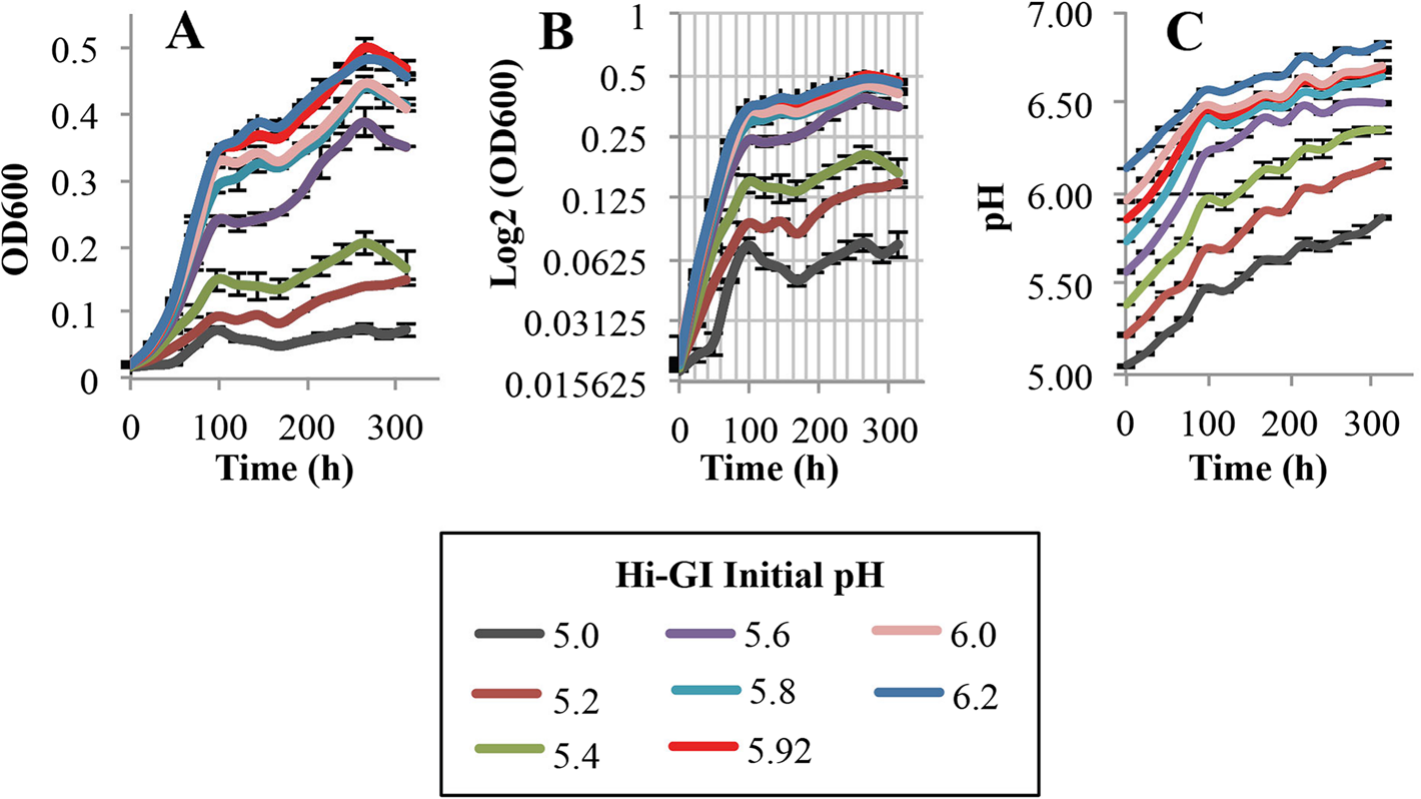
*L. crescens* growth with pH at a range of initial pH (5.0–6.2) in Hi-GI medium. *L. crescens* was grown to exponential phase in Hi-GI at 150 rpm, 28 °C (OD_600_~0.5–0.6, Fig. 15) and washed with a sterile ACES-KOH buffer pH 6.5 prior to inoculation. Optimal growth was achieved between the pH range of 5.8 and 6.2. **a** Cell growth represented as optical density, **b** Cell growth in Logarithmic (log2) scale of the optical density, and **c** pH of the external medium over time during cell growth

**Fig. 4:**
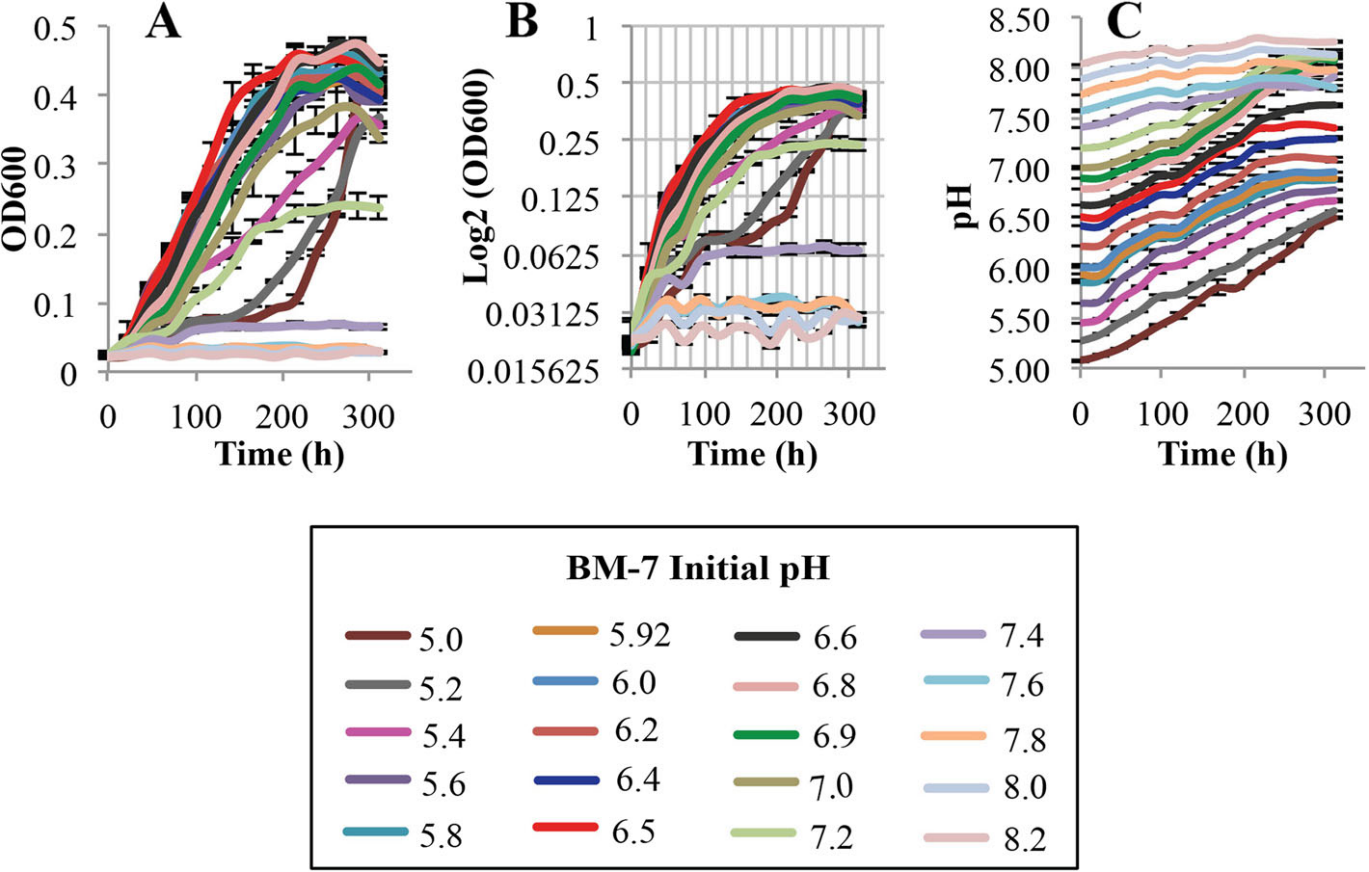
*L. crescens* growth with pH at a range of initial pH (5.0–8.2) in BM-7 medium. *L. crescens* was grown to exponential phase in Hi-GI at 150 rpm, 28 °C (OD_600_~0.5–0.6, Fig. 15) and washed with a sterile ACES-KOH buffer pH 6.5 prior to inoculation. Optimal growth was observed between pH 5.8 and 6.8. **a** Cell growth represented as optical density, **b** Cell growth in Logarithmic (log2) scale of the optical density, and **c** pH of the external medium over time during cell growth

The first variable in the evaluation of growth parameters was optimal pH for the growth of *L. crescens* in the M15, Hi-GI, and BM-7 media. The pH evaluated in each medium also includes that which is found in the citrus phloem (5.0–5.74) where the citrus pathogen Ca. *L. asiaticus* resides [11]. The pH range evaluated for each medium was also limited by the ability of the media to remain in solution. Thus, the pH ranges evaluated were as follows: M15, pH 5.0–7.0; Hi-GI, pH 5.0–6.2; and, BM-7, pH 5.0–8.2 with 0.2 increments in each of the media (Figs. 2, 3, 4; see Additional file 1). Growth (OD_600_) and pH were simultaneously monitored in the cultures over a 13 day period (Figs. 2, 3, 4; see Additional file 1).

In M15 chemically-defined medium, *L. crescens* grew best at pH 5.92 compared to the other initial pH tested (Tukey HSD *p* < 0.001), with exception of pH 6.0 (Tukey HSD, *p* = 0.31) (Fig. 2; see Additional file 1) However, the growth is slightly better in M15 pH 5.92 compared to M15 pH 6.0 (Fig. 2; see Additional file 1). The optimal pH range in Hi-GI encompassed 5.8 to 6.2, where *L. crescens* grew similarly (Tukey HSD, 0.65 < *p* < 0.99); whereas in the other pH, the growth was significanlty reduced (Tukey HSD, *p* < 0.001) (Fig. 3; see Additional file 1). In BM-7, the optimal growth was between pH 5.8 and 6.8 (Tukey HSD, 0.99 < *p* < 1), followed by pH 6.9, 5.6, and 7.0 (Tukey HSD, 0.4 < *p* < 0.99) (Fig. 4; see Additional file 1). The growth of *L. crescens* in BM-7 with a pH lower than 5.6 was significantly reduced compared to the pH range between 5.6 and 7.0 (Tukey HSD, *p* < 0.001), requiring longer time to enter in exponential phase (Fig. 4; see Additional file 1). In BM-7 media with pH higher than 7.0, *L. crescens* grows poorly (Tukey HSD, *p* < 0.001) (Fig. 4; see Additional file 1). Strong positive spearman rho and pearson linear correlations were found between optical density and external pH in M15, Hi-GI, and BM-7 (see Additional file 2). Since *L. crescens* grew poorly in BM-7 media with a pH higher than 7.4, the correlations between pH and growth were also small. (Fig. 4, pH 7.6 to 8.2; see Additional file 1). An increase in pH alongside an increase in optical density was evident in all media (Figs. 2, 3, 4; see Additional file 1).

*L. crescens* grew optimally at an initial pH ranging from 5.8 to 6.8 in axenic culture. Ideal growth was achieved at pH 5.92 for M15 and Hi-GI media, whereas in BM-7, ideal growth was obtained at pH 6.5. In the media with a pH lower than 5.8, *L. crescens* grew poorly, requiring more time to start exponential growth. At this lower pH, *L. crescens* still induced a pH increase in the media, which might reflect the ability of the cells to survive and adapt to acidic conditions, even at low titers. The limitations to culture Ca. *L. asiaticus* might be reflected in the inability to keep higher titers within the acidic phloem sap [11, 27].

### Second step: determining aeration levels for the growth of *L. crescens*

**Fig. 5:**
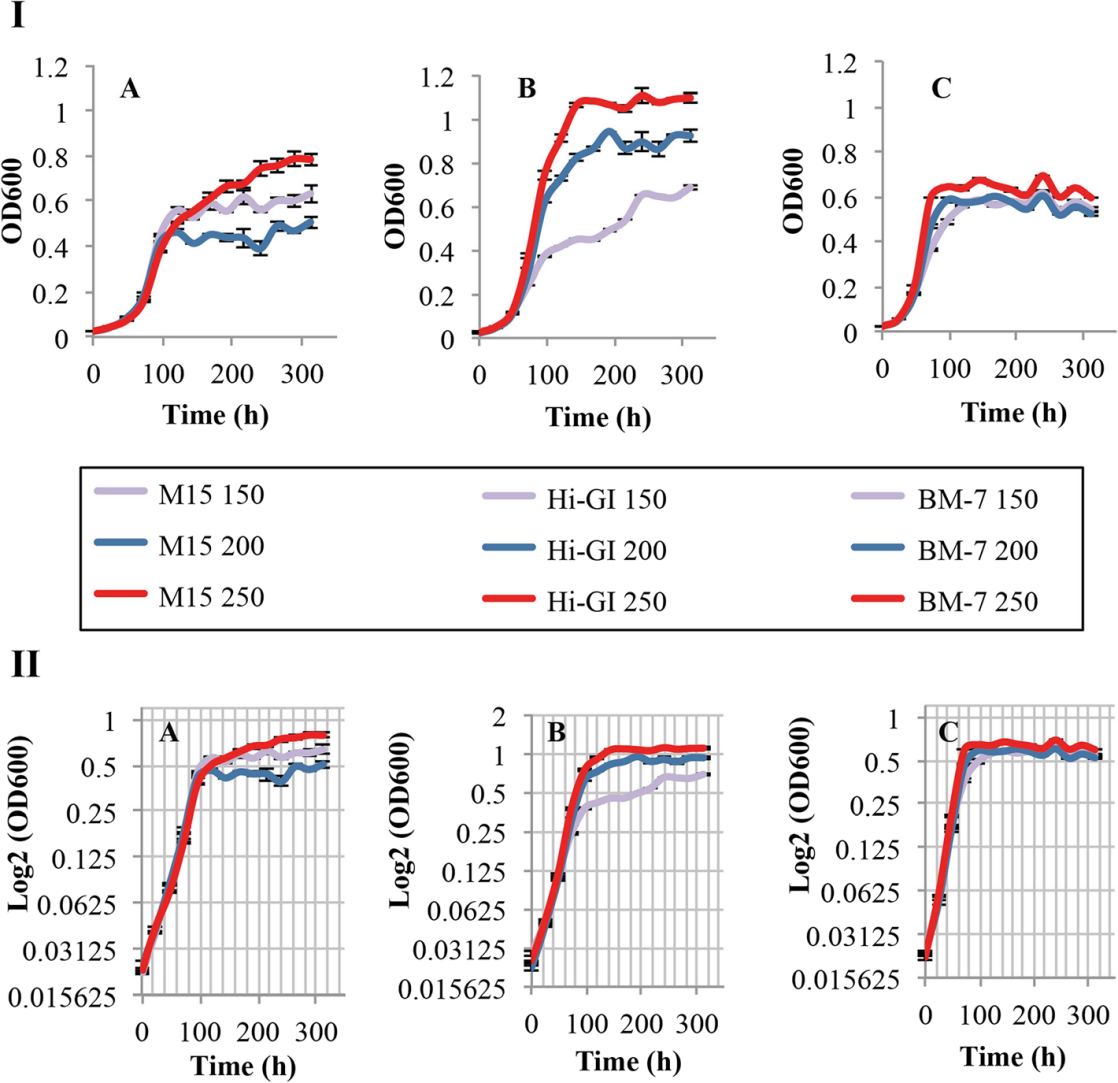
*L. crescens* growth at different shaking speeds over a 13-day period. *L. crescens* was grown to exponential phase in Hi-GI at 150 rpm, 28 °C (OD_600_~0.5–0.6, Fig. 15) and washed with a sterile ACES-KOH buffer pH 6.5 prior to inoculation. Growth was observed in M15 at 250 rpm and in Hi-GI medium at 200 and 250 rpm. No growth improvements were observed in BM-7 medium. I) Cell growth represented as optical density. II) Cell growth in Logarithmic (log2) scale of the optical density. **a** M15, pH 5.92, **b** Hi-GI, pH 5.92, and **c** BM-7, pH 6.5

The second variable in the evaluation of growth parameters was the aeration requirements. *L. crescens* is an aerobic bacterium that utilizes oxygen as the final electron acceptor in the electron transport chain throughout aeration [8, 28]. Thus far, *L. crescens* only grows at 150 rpm in axenic culture, and no optimization has yet been performed to evaluate the optimal aeration requirement levels for growth [7, 8, 10]. Here, three growth conditions—50, 200, and 250 rpm—at optimal pH were evaluated for the growth of *L. crescens* in axenic culture. Better growth is observed in M15 250 rpm and Hi-GI 200/250 rpm compared to 150 rpm (Tukey HSD, *p* < 0.001) (Fig. 5a,b; see Additional file 1). None of the aeration conditions improved the growth of *L. crescens* in BM-7 compared to the 150 rpm control (Tukey HSD, *p* > 0.05) (Fig. 5c; see Additional file 1). However, the growth curve at 250 rpm showed a smoother line compared to 150/200 rpm for all three media. Thus, 250 rpm was used as the optimal aeration condition in the subsequent step.

### Third step: optimal temperature for growth of *L. crescens*

**Fig. 6:**
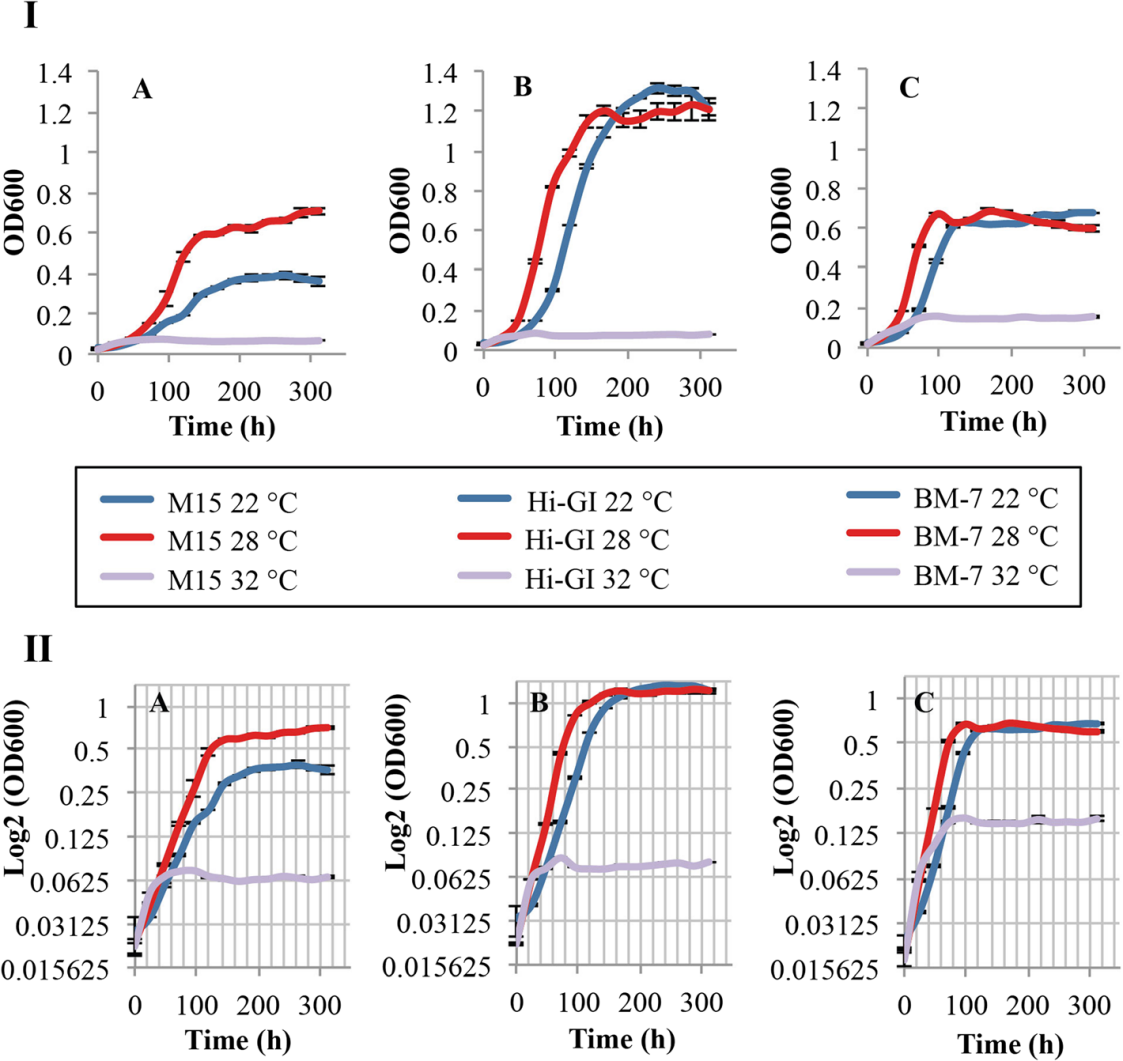
*L. crescens* growth at different temperatures with optimal pH and aeration requirements (250 rpm) constant. *L. crescens* was grown to exponential phase in Hi-GI at 250 rpm, 28 °C (OD_600_~0.7–0.8, Fig. 16) and washed with a sterile ACES-KOH buffer pH 6.5 prior to inoculation. Better growth is observed in M15 medium at 28 °C compared to 22 °C. *L. crescens* growth is similar in Hi-GI and BM-7 media at 22 °C and 28 °C. No growth is reported at 32 °C in the three media. I) Cell growth represented as optical density. II) Cell growth in Logarithmic (log2) scale of the optical density. **a** M15, pH 5.92, **b** Hi-GI, pH 5.92, and **c** BM-7, pH 6.5

The third variable in the evaluation process for *L. crescens* growth was temperature. To date, *L. crescens* has only been cultured at 27/28 °C in axenic culture media [4, 7, 8, 10]. Here, *L. crescens* growth was evaluated at three different temperatures (22, 28 and 32 °C) at optimal pH and 250 rpm. These temperatures were chosen based on reports on Ca. *L. asiaticus* and Ca. *L. americanus* growth temperatures in citrus phloem [15]*.* In M15, better growth was observed at 28 °C compared to 22 °C (Tukey HSD, *p* < 0.000) (Fig. 6a; see Additional file 1). *L. crescens* grew well and statistically similar at 28 °C and 22 °C in Hi-GI and BM-7 over a 13-day period (Tukey HSD, 0.56 < *p* < 0.64) (Fig. 6b,c; see Additional file 1). No growth was observed at 32 °C in the three media (Fig. 6; see Additional file 1).

### Fourth step: the buffering requirements for *L. crescens* growth

The fourth variable evaluated for *L. crescens* growth was the ability of buffers to prevent the pH increase of the medium over time, in hopes that this would improve culture growth. The buffers tested include MES (pKa = 6.2), Bis-Tris hydrochloride (pKa = 6.5), ACES (pKa = 6.8), and monosodium phosphate (pKa = 7.2) at 10, 25 and 50 mM concentrations. ACES buffer is present in the BM-7 at 54.3 mM, whereas monosodium phosphate buffer is present in the M15 at 11.69 mM and in Hi-GI medium at 8.4 mM.

**Fig. 7:**
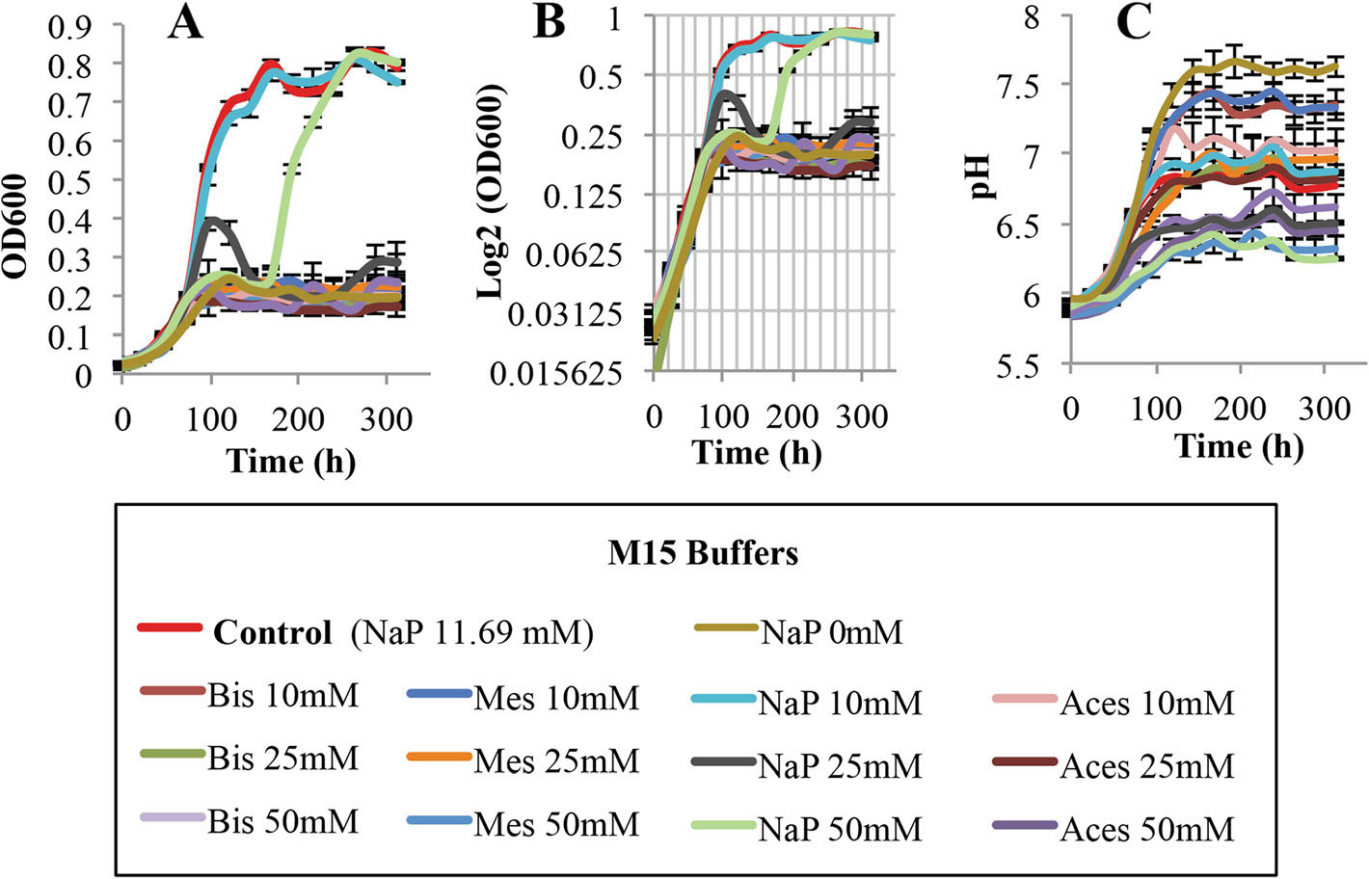
Growth and pH assessments of *L. crescens* cultures in M15 with four biological buffers. These buffers include Monosodium phosphate (NaP), ACES, Bis-Tris HCl (Bis), and MES at 10, 25 and 50 mM concentrations. *L. crescens* was grown to exponential phase in Hi-GI at 250 rpm, 28 °C (OD_600_~0.7–0.8, Fig. 16) and washed with a sterile ACES-KOH buffer pH 6.5 prior to inoculation in M15 with the different buffers at an initial pH of 5.92. Only phosphate buffer 10 mM promotes the growth of *L. crescens* in a similar rate to that of the M15 control. Increase of the external pH was evident in all the media, even at 50 mM concentrations of the buffers. **a** Cell growth represented as optical density, **b** Cell growth in Logarithmic (log2) scale of the optical density, and **c** pH of the external medium over time during cell growth

**Fig. 8:**
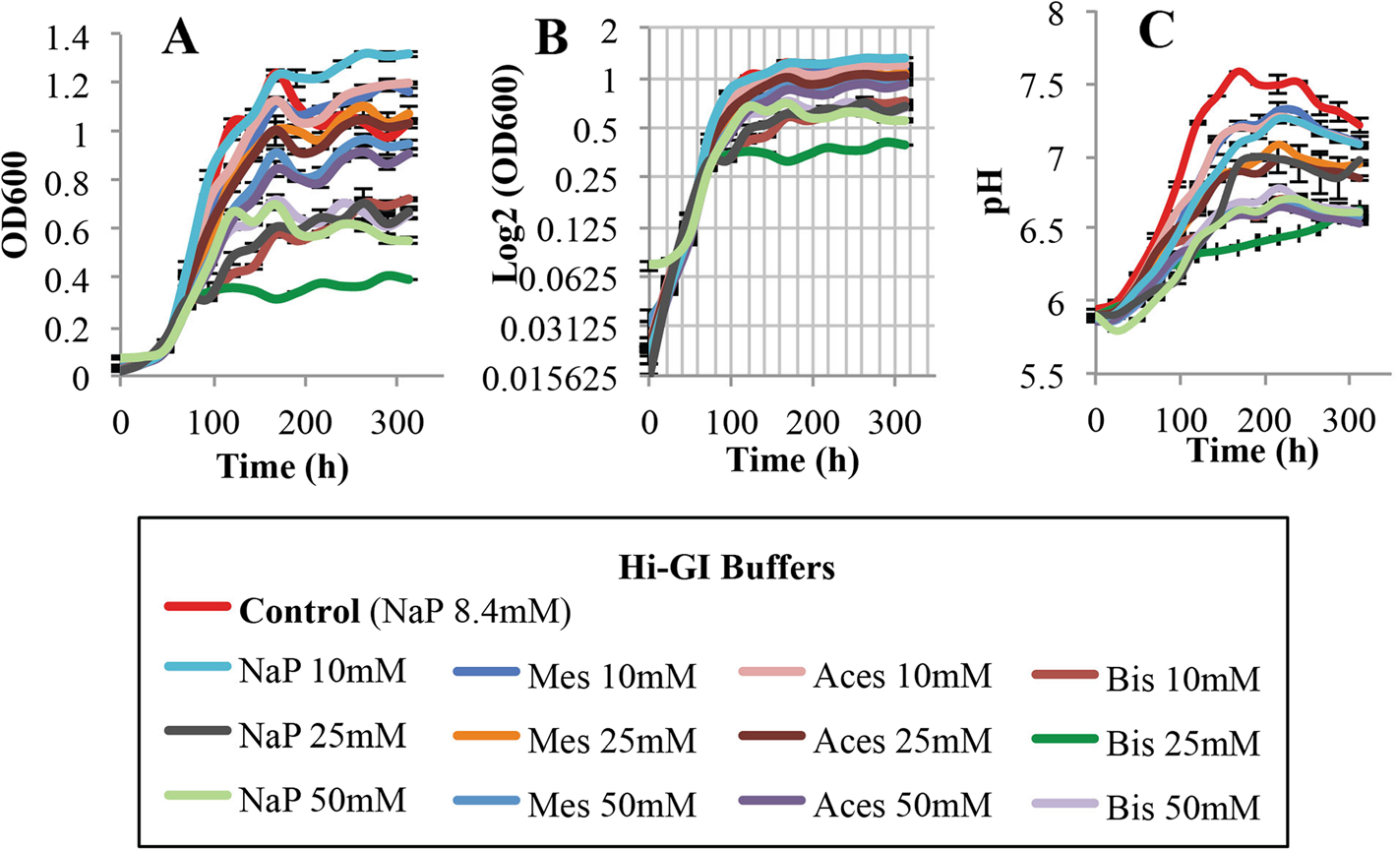
Growth and pH assessments of *L. crescens* cultures in Hi-GI medium using four biological buffers. These buffers include Monosodium phosphate (NaP), ACES, Bis-Tris HCl (Bis), and MES at 10, 25 and 50 mM concentrations. *L. crescens* was grown to exponential phase in Hi-GI at 250 rpm, 28 °C (OD_600_~0.7–0.8, Fig. 16) and washed with a sterile ACES-KOH buffer pH 6.5 prior to inoculation in Hi-GI with the different buffers at an initial pH of 5.92. Phosphate, MES and Aces at 10 mM promoted the growth of *L. crescens* at similar rates to those of the Hi-GI control. Increase of the external pH was evident in all the media, even at 50 mM concentrations of the buffers. **a** Cell growth represented as optical density, **b** Cell growth in Logarithmic (log2) scale of the optical density, and **c** pH of the external medium over time during cell growth

**Fig. 9:**
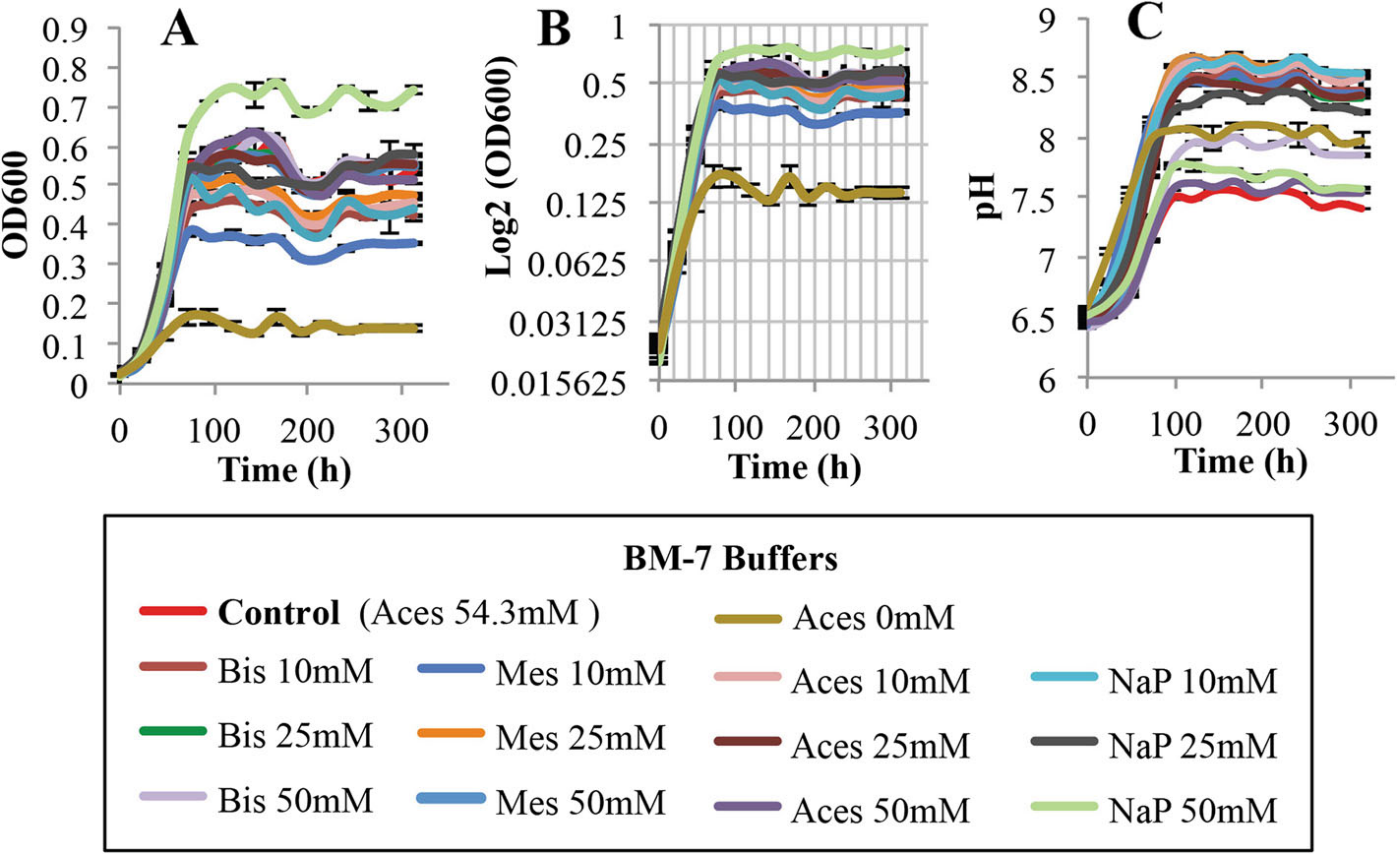
Growth and pH assessments of *L. crescens* cultures in BM-7 medium using four biological buffers. These buffers include Monosodium phosphate (NaP), ACES, Bis-Tris HCl (Bis), and MES at 10, 25 and 50 mM concentrations. *L. crescens* was grown to exponential phase in Hi-GI at 250 rpm, 28 °C (OD_600_~0.7–0.8, Fig. 16) and washed with a sterile ACES-KOH buffer pH 6.5 prior to inoculation in BM-7 with the different buffers at an initial pH of 6.5. The growth of *L. crescens* is similar in all media, except for phosphate at 50 mM and MES 10 mM with better and worse growth, respectively. No growth is observed in the BM-7 medium without buffer. pH increase with all the buffers is similar or higher than the BM-7 control. **a** Cell growth represented as optical density, **b** Cell growth in Logarithmic (log2) scale of the optical density, and **c** pH of the external medium over time during cell growth

All buffers tested in M15 medium, except for monosodium phosphate buffer 10 mM, reduced growth of *L. crescens* compared with the M15 control (Tukey HSD, *p* < 0.001) (Fig. 7a,b; see Additional file 1). Only monosodium phosphate (10 mM), ACES (10, 25 mM), and MES (10, 25 mM) buffers promoted growth at the same rates as those seeing in Hi-GI control (Tukey HSD, *p* = 1) (Fig. 8a, b; see Additional file 1). None of the buffers in BM-7 increased *L. crescens* growth over that provided by the BM-7 control (Tukey HSD, *p* > 0.05). They also do not maintain a constant pH (Fig. 9a, b; see Additional file 1). In the three media, monosodium phosphate 10 mM appeared to be suitable for the optimal growth of *L. crescens* at similar rates to that of the controls. Moderate to strong positive Spearman and Pearson correlations were observed between pH increase and the growth of *L. crescens* with most of the buffer concentrations (see Additional file 3). Only 50 mM concentration of the buffers in M15 and Hi-GI media reduced the pH increase of the external media, but these treatments severely affected growth (Figs. 7c and 8c; see Additional file 1). In BM-7 medium, 50 mM concentration of the buffers neither affects the growth nor the pH increase compared with the BM-7 control (Fig. 9; see Additional file 1).

The inability of the buffers to control the increase of pH suggests that *L. crescens* posses an endogenous mechanism to maintain pH homeostasis inside the cell. In the slightly acidic environment in which *L. crescens* grows, the influx of H^+^ protons to the cell might contribute to generate proton motive force (PMF) [29]. However, excess proton uptake might also impair pH homeostasis in the cytoplasmic space [30]. Therefore, excess of protons inside and outside the cell requires to be controlled by a pH regulatory mechanism [30], which can guarantee the proper growth of *L. crescens* in slightly acidic conditions.

### Optimal phosphate levels for the growth of *L. crescens*

**Fig. 10:**
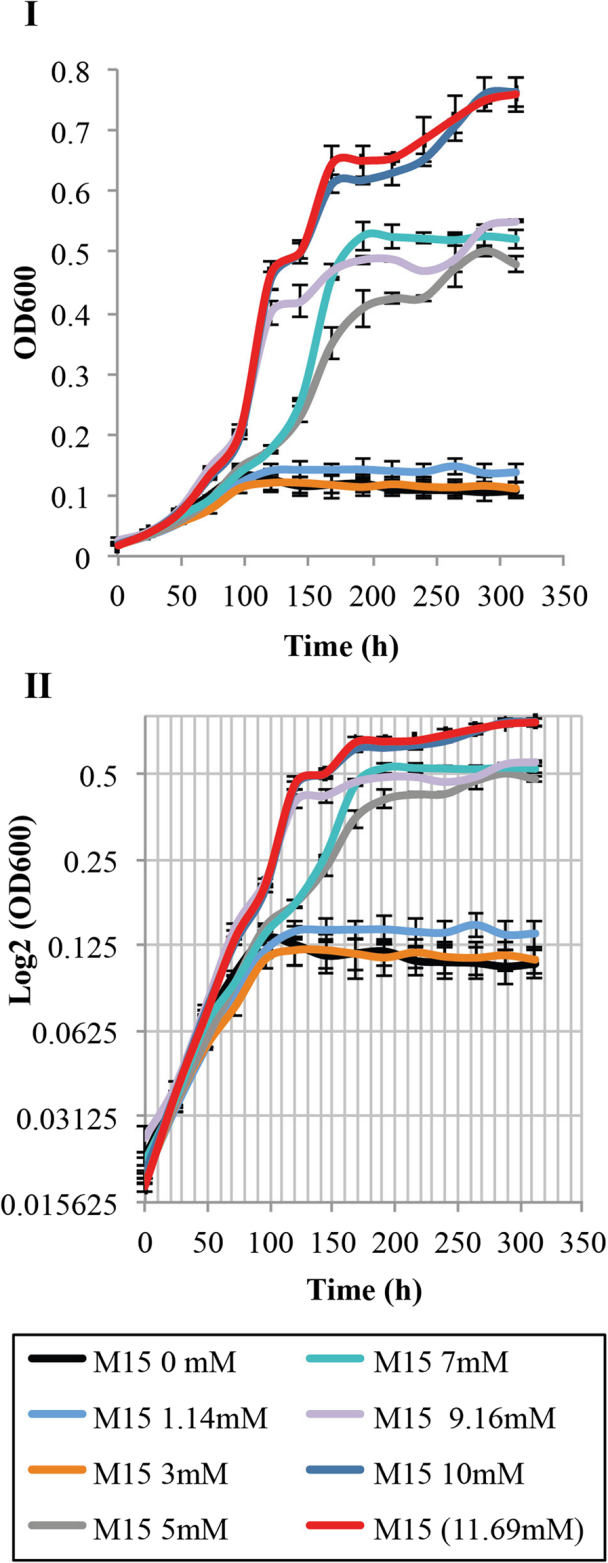
Phosphate requirements for *L. crescens* growth using phosphate levels found in phloem of citrus cultivars [11]. *L. crescens* was grown to exponential phase in Hi-GI at 250 rpm, 28 °C (OD_600_~0.7–0.8, Fig. 16) and washed with a sterile ACES-KOH buffer pH 6.5 prior to inoculation in the M15 medium, pH 5.92. Best growth of *L. crescens* was observed in 10 mM and 11.69 mM (M15 control) concentrations of monosodium phosphate. I) Cell growth represented as optical density. II) Cell growth in Logarithmic (log2) scale of the optical density

Since phosphate was the preferred buffer for the growth of *L. crescens* in axenic culture, the optimal phosphate requirements were investigated using the concentrations found in citrus phloem (1.14 mM to 9.16 mM) [11]. Better growth was observed with phosphate concentrations equal to and higher than 5 mM, with optimal growth observed at 10 mM and 11.69 mM (Tukey HSD, *p* = 1) (Fig. 10; see Additional file 1). Little to no growth is observed with concentrations lower than 5 mM of phosphate (Fig. 10; see Additional file 1).

### pH modulation in *L. crescens* cultures in the M15 chemically-defined medium

To further investigate the cause of the pH increase during growth of *L. crescens* cultures, three potential phenomena associated with pH homeostasis within the cell were studied in the M15 chemically-defined medium: citrate metabolism, ornithine metabolism, and ammonium accumulation in the external media.

**Fig. 11:**
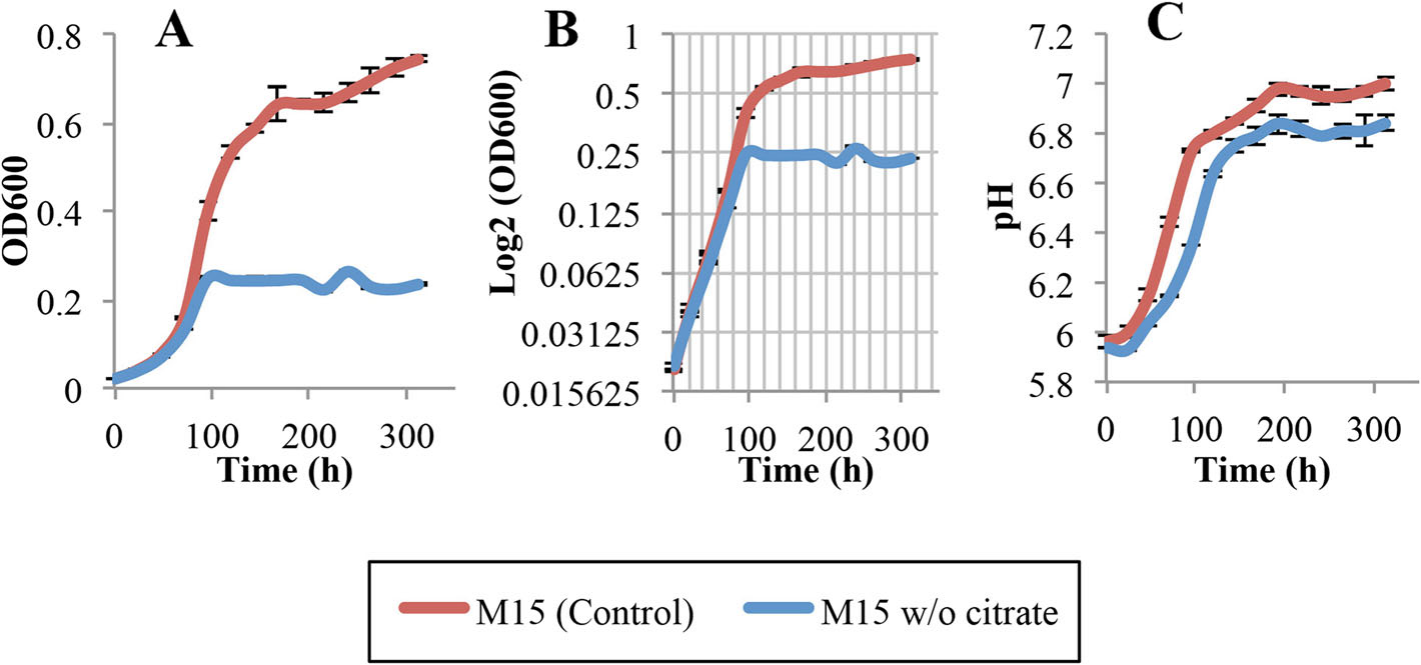
Growth and external pH of *L. crescens* cultures growing in M15 and M15 without citrate. *L. crescens* was grown to exponential phase in Hi-GI at 250 rpm, 28 °C (OD_600_~0.7–0.8, Fig. 16) and washed with a sterile ACES-KOH buffer pH 6.5 prior to inoculation. Removal of citrate to the M15 medium significantly reduced the growth of *L. crescens,* without affecting the increase of the external pH. **a** Cell growth represented as optical density, **b** Cell growth in Logarithmic (log2) scale of the optical density, and **c** pH of the external medium over time during cell growth

First, each citrate molecule may be imported into the cell along with a proton, thereby increasing the pH of the external medium [16, 17, 30]. *L. crescens* uptakes the abundant citrate in the M15 medium in a H^+^ symport dependent manner through a yet-unknown transporter [10, 31]. One possibility is that the *Liberibacter* homolog of the C_4_-dicarboxylate transport system in *Sinorhizobium meliloti* can transport citrate as well as C_4_-organic acids [10, 31]. However, citrate transport in *Liberibacter* needs to be studied in more detail. Omission of citrate from the medium greatly reduced *L. crescens* growth [10], but it does not affect the increase of pH compared with M15 (Tukey HSD, *p* = 0.77) (Fig. 11; see Additional file 1). Therefore, removal of citrate from M15 is not responsible for the pH increase in *L. crescens* cultures.

**Fig. 12:**
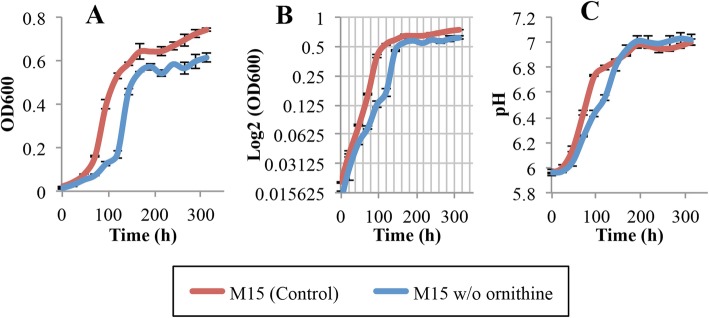
Growth and external pH of *L. crescens* cultures growing in M15 and M15 without ornithine. *L. crescens* was grown to exponential phase in Hi-GI at 250 rpm, 28 °C (OD_600_~0.7–0.8, Fig. 16) and washed with a sterile ACES-KOH buffer pH 6.5 prior to inoculation. Removal of ornithine affects neither the growth nor the pH increase of the medium compared with the M15 control. **a** Cell growth represented as optical density, **b** Cell growth in Logarithmic (log2) scale of the optical density, and **c** pH of the external medium over time during cell growth

Alternatively, ornithine decarboxylase in *Liberibacter* may decarboxylate ornithine to putresine, thereby increasing pH upon export [32]. However, omitting ornithine from M15 did not reduce the increase in pH with *L. crescens* growth compared with M15 (Tukey HSD, *p* = 0.12) (Fig. 12; see Additional file 1). The removal of ornithine alone does not affect the growth of *L. crescens* compared to M15 control (Fig. 12; see Additional file 1).

### Ammonium accumulation of *L. crescens* cultures

**Fig. 13:**
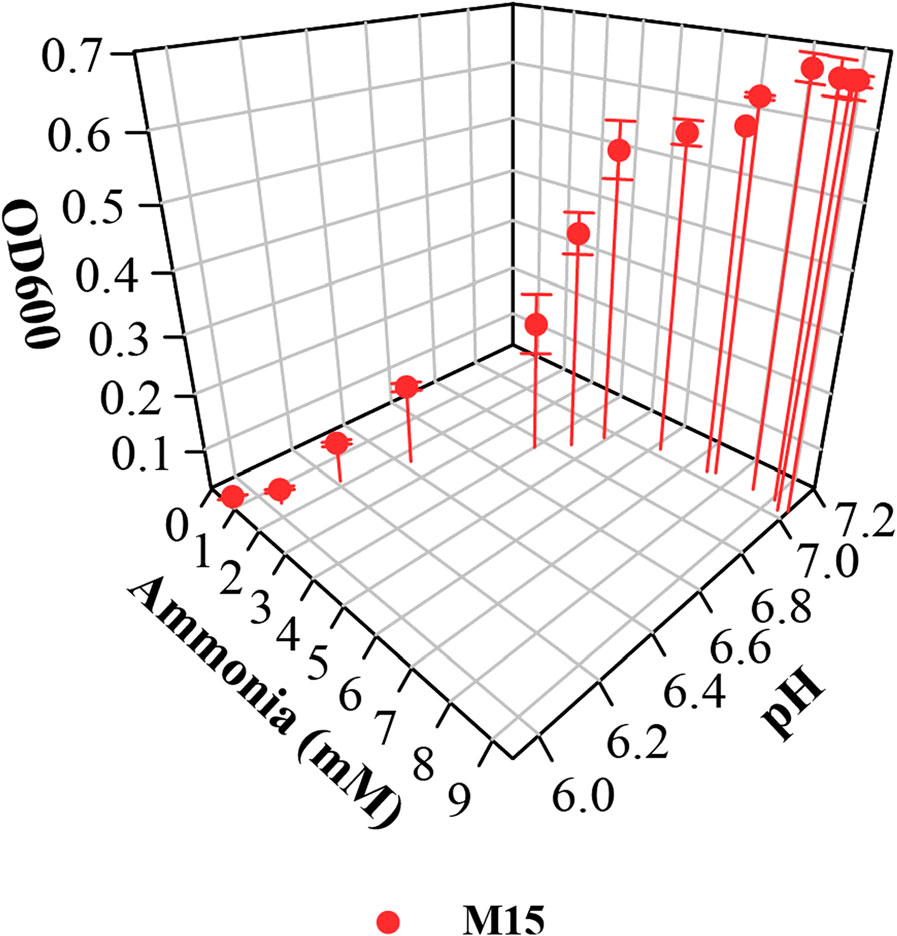
Accumulation of ammonia in M15 cell-free supernatant of *L. crescens* cultures over a 13-day period. *L. crescens* was grown to exponential phase in Hi-GI at 250 rpm, 28 °C (OD_600_~0.7–0.8, Fig. 16) and washed with a sterile ACES-KOH buffer pH 6.5 prior to inoculation. Concentration of ammonia/ammonium in the cell-free supernatant increased from 0.611 to 9.47 mM along with bacterial growth and pH increase of M15 medium

**Fig. 14:**
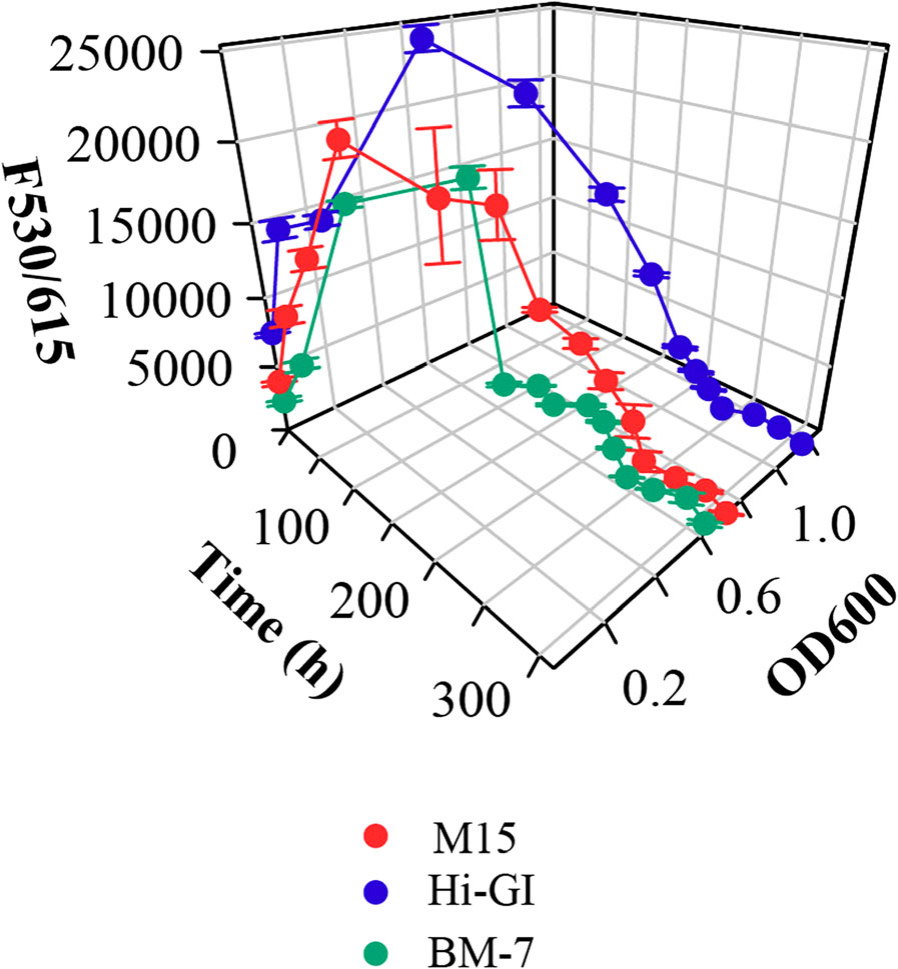
Viability of *L. crescens* cells using Presto Blue reagent in M15, Hi-GI and BM-7. *L. crescens* was grown to exponential phase in Hi-GI at 250 rpm, 28 °C (OD_600_~0.7–0.8, Fig. 16) and washed with a sterile ACES-KOH buffer pH 6.5 prior to inoculation. Maximal viability of the cells is observed between 72 and 96 h of growth in the different media

A third possibility is that ammonia accumulation in M15 occurs during *L. crescens* culture growth. In M15 medium, concentrations of amino acids vary between 0.11 and 11.45 mM [10]. Thus, amino acid deamination may be the source of ammonia that is increasing the pH of the medium over time [29]. The resulting α-keto acids might then be used as energy sources while excess ammonium is transported outside of the cell by diffusion or active transport inducing alkalization of the external medium [33]. Hence, the first question to ask is whether ammonia is exported into the medium during cell culture growth and whether levels of ammonia in the medium were correlated with pH increase. Ammonia/ammonium levels did increase with the growth of *L. crescens* and these levels were positively correlated with increasing pH (Fig. 13, Table 1; see Additional file 1). The concentration of ammonia/ammonium increased from 0.61 mM at 0 h to 4.57 mM at 120 h during maximal cell viability at exponential and stationary growth, to 9.47 mM at 312 h when maximum cell death is reached (Figs. 13 and 14; see Additional file 1). Therefore, excess ammonium appears to responsible for the increase of pH in the spent M15 medium of *L. crescens* cultures.

**Table 1:** Spearman rank correlation and Pearson lineal correlation of *L. crescens* cultures in M15 medium. Correlations were established among the concentration of ammonia, the increase of pH and the growth (OD_600_) of *L. crescens*. The three variables are positively and highly correlated with correlation coefficients ranging from 0.88 to 0.96

	Spearman Rho (R)	Spearman (*p* value)	Pearson (R)	Pearson (*p* value)
pH-ammonia	0.883042756	1.02141E-14	0.957298542	1.33E-15
OD_600_-pH	0.923691063	0	0.957298542	0
OD_600_-Ammonia	0.953836178	0	0.953463717	0

### Cell viability in the optimal growth conditions of *L. crescens*

In order to provide more accurate information about the period required for the growth of *L. crescens*, the viability of the cells was monitored in the three optimized media using the Presto Blue viability reagent assay. The reducing ability of living cells causes rezasurin—the active ingredient of presto blue—to be reduced to resorufin, a highly fluorescent compound [34]. Thus, the amount of fluorescence is proportional to the living cells present in the media. Maximal viability of the cells is achieved between 72 to 96 h for M15 and Hi-GI, and 48 to 72 h for BM-7 (Fig. 14; see Additional file 1). The fluorescence levels observed are representative of the growth curves, where Hi-GI medium has the highest peak viability level, followed by M15 and BM-7 media. A gradual cell death starts at 120 h in M15 and Hi-GI while an abrupt live-cell decline is found in BM-7 at 96 h (Fig. 14; see Additional file 1). Optical density is simultaneously monitored in the same cultures to correlate with cell viability over time (Fig. 14; see Additional file 1).

## Discussion

The evaluation of the growth parameters of *L. crescens* reveals the lifestyle of the Ca. Liberibacter pathogens. The evaluation strategy showed that *L. crescens* grows optimally in axenic culture at a pH between 5.8–6.8, at 28 °C, with phosphate as the preferred buffer for growth. The citrus phloem in which Ca. *L. asiaticus* resides has a pH of 5.0 to 5.74 [11]. In this acidic pH, *L. crescens* struggles to grow, requiring more time to enter into the exponential phase. The low titers of Ca. *L. asiaticus* in the citrus phloem might be associated with the acidic conditions this pathogen faces prior to colonization [27]. Thus, *Liberibacter* species appear to be neutrophilic bacteria that adapted to slightly acidic environments. Similar to *L. crescens* optimal growth temperature (27/28 °C), Ca. *L. asiaticus* and Ca. *L. americanus* have also reported optimal growth in the citrus phloem at 22/27 °C night/day photoperiod [15].

None of the buffers improved the growth of *L. crescens* compared to the controls. However, monosodium phosphate buffer at 10 mM promoted the growth of *L. crescens* at similar rates to those of the three media controls. Phosphate buffer is commonly used in the growth of neutrophilic bacteria, not only to maintain pH, but as a source of phosphorous. Phosphate is present in the phloem of different citrus cultivars at concentrations between 1.14 and 9.16 mM [11]. It is also present in the haemolymph of *D. citri* at a concentration of 4.77 mM per psyllid haemolymph [12]. Substantial growth of *L. crescens* was observed with phosphate buffer at concentrations between 5 mM and 11.69 mM in M15 medium. Optimal growth was observed at 10 mM and 11.69 mM. Higher concentrations than 11.69 mM phosphate should be carefully considered because phosphate and citrate together, in a medium rich in calcium, induce calcium phosphate precipitation, leading to erroneous results [35]. Since HLB-affected plants have been reported to be deficient in phosphorous [36], it is likely that Ca. *L. asiaticus* also requires phosphorous in the form of phosphate to survive within the hosts. However, the phosphorous-deficient HLB-affected citrus plants might also require the spray of phosphate in concentrations lower than 3 mM to improve the health of the trees and to reduce Ca. *L. asiaticus* titers [36]. Thus, phosphate might play an important role in reducing disease symptoms.

The increase of pH during culture growth, even using different buffers, can be explained by the accumulation of ammonium in milimolar concentrations in the external medium of *L. crescens* cultures. In *Arabidopsis thaliana,* the production of ammonium even at micromolar concentrations by several strains of rhizosphere bacteria induces plant growth inhibition and chlorosis [19, 37]. The citrus phloem and psyllid haemolymph are rich in amino acids with concentrations ranging from 0.16 to 44.9 mM per citrus phloem sample and 0.008 to 0.75 mM per psyllid haemolymph sample [11, 12]. In the rich amino acid phloem environment, Ca. *L. asiaticus* might also deaminate amino acids resulting in the accumulation of ammonium in the phloem. In the citrus plants, excess ammonium could further induce ionic and pH disturbances as well as oxidative stress. These events lead to leaf chlorosis and stunted roots and shoots, the common symptoms of HLB-disease [19, 20].

The deamination activity in *L. crescens* is not well understood. All *Liberibacter* species contain glutaminase, which deaminates glutamine to glutamate and ammonia [38]. *L. crescens* and Ca. *L. asiaticus* have NAD-glutamate dehydrogenase, which converts L-glutamate to α-ketoglutarate and ammonia [39]. However, preliminary studies show that none of these enzymes is responsible for ammonium release and the pH increase in the external media of *L. crescens* (data not shown)*.* Other worth-considering alternative is the presence of two nucleoside deaminases in *L. crescens* and Ca. *L. asiaticus*, which catalyze the hydrolytic deamination of cytidine and adenosine to uridine and inosine with the concomitant production of ammonia [40]. However, the activity of these nucleoside deaminases is not expected to generate the high concentrations of ammonium found in *L. crescens* culture media. Improvements in the functional annotation of *Liberibacter* genomes could lead to better identify ammonia-producing enzymes in these bacteria.

Since excess ammonium is toxic for the plants, its uptake and assimilation has to be tightly regulated through saturable high affinity (ammonium transporters AMTs) and non-saturable low affinity uptake systems (aquaporins or cation channels) [41]. In citrus plants, ammonium uptake in the roots is regulated by light and sucrose availability in the roots. Illumination in the shoots stimulates photosynthesis with the concomitant production of soluble photosynthates such as sucrose, glucose, fructose and organic acids [21]. These photosynthates, especially sucrose, are transported via phloem to the roots, to facilitate the high affinity uptake of ammonium through the CitAMT1 transporter. Sucrose acts as signal molecule that coordinates between N uptake systems and C activity in the roots [21]. Carbon availability and reducing equivalents are necessary to induce ammonium uptake and assimilation in the roots [42]. In plants with limited C root assimilatory capabilities, ammonium is loaded into the xylem and subsequently assimilated into sink compartments such as mesophyll cells and companion cells [42, 43]. The phloem is the main transport system of sugars, carboxylic acids and amino acids from source to sink growing tissues in the shoots and in the roots [18]. Ammonium can be assimilated in the phloem companion cells using the glutamine synthetase/glutamate synthase (GS/GOGAT) system or the glutamate dehydrogenase (GDH) [44, 45]. However, ammonium is not loaded into the phloem [46], for which over-accumulation of ammonium in the phloem sieve tubes by pathogen activity could lead to nitrogen (N) imbalances with toxic effects to the citrus plants.

Strategies to regulate the accumulation of ammonium in the phloem are not well studied yet. However, several ways to lower excess ammonium in the plant as a whole have been reported. For instance, increasing the light intensity to wheat plants and citrus plants can induce a higher ammonium accumulation rate to amino acids and proteins in the roots due to higher tricarboxylic acid loading and the activation of GDH [21, 25]. The application of exogenous carbon supplies such as sucrose in to citrus and calcium carbonate (CaCO_3_) to cucumber provides with additional carbon (C) skeletons for NH4^+^ assimilation in the roots [21, 22]. The addition of increased concentrations of K^+^ reduces ammonium flux to the roots by active competition for transport through K^+^ specific channels in rice [23]. Low doses of nitrate can also contribute to reduce ammonium toxicity since NO_3_^−^ uptake induces alkalization of the medium to counterbalance the effect of NH4^+^ [24, 26]. Finally, the addition of phosphate to the roots of *A. thaliana* induces ammonium phosphate salt precipitates restoring plant growth [37]. If Ca. *L. asiaticus* growth contributes to the accumulation of ammonium in the citrus phloem, the interplay between ammonium and these ions and molecules could contribute to alleviate HLB-symptoms. However, these strategies need to be studied in more detail. The molecular mechanisms of deamination in *Liberibacter* species, the accumulation of ammonium in the citrus phloem, the potential effects of the different strategies to reduce the ammonium levels in the phloem of citrus plants as well as more thorough statistical methods to evaluate growth optimization parameters (E.g. Surface Response Methodology and Placket-Burman Plot) are still subjects of investigation.

## Conclusions

The evaluation strategy for the growth of *L. crescens* opens new avenues to understand the lifestyle of *Liberibacter* species and the potential interactions within the hosts. *L. crescens* grows in slightly acidic conditions, at 28 °C, in a fully oxygen environment and phosphate as the preferred buffer for growth. Ca. Liberibacter pathogens are expected to grow in similar environmental conditions to those of *L. crescens* because they are metabolically similar and grow in similar environments—the phloem sap and their insect vectors. In Ca. *L. asiaticus*, different factors such as the slightly acidic citrus phloem, the repertory of enzymes for aerobic respiration, the optimal growth temperatures within the phloem, and the phosphoric acid present in the hosts highlight these similarities. The accumulation of ammonium is the main driver of pH modulation in *Liberibacter* species. In citrus plants, excess ammonium is expected to induce ionic stress, pH imbalances, and disturbances to the host cell membranes, with the concomitant development of HLB-disease symptoms. Studying different strategies to reduce ammonium in the citrus phloem might lead to reduce symptoms in HLB-affected trees.

## Methods

### Reagents, bacterial strains, and culture conditions

TNM-FH insect medium and Fetal Bovine Serum (FBS) were purchased through Gemini Bio-products (Sacramento, CA). Hi Grace’s Insect Medium (Hi-GI) was purchased through Hi-Media (Mumbai, India). Citric acid was purchased through Fisher Scientific (Pittsburgh, PA), L-arginine hydrochloride through Calbiochem (San Diego, CA), L-lysine monohydrochloride through Acros-organics (Geel, Belgium), and methionine sulfoxide through Alfa Aesar (Haverhill, MA). All other reagents were obtained through Sigma-Aldrich (St. Louis, MO), unless specified. BM-7 and M15 media were prepared as described previously [8, 10]. For M15 preparation, most components were dissolved with gentle stirring, except for L-tyrosine and three non-water soluble vitamins—biotin, folic acid and riboflavin. L-tyrosine was dissolved in 15 ml of 2 mM HCl prior to addition to M15. Biotin was dissolved in dimethyl sulfoxide, folic acid in 0.1 M NaOH, and riboflavin in 0.01 M NaOH. DL-serine of M15 was replaced by L-serine. Hi-Gi medium was prepared according to the manufacturer instructions. Briefly, 45.9 g of Hi-GI were dissolved in 950 mL of sterile, deionized water, slowly adjusting the pH to 5.92 by titration with 5 M KOH. After completing to 1 L, Hi-GI was sterilized through a 0.22 μm sterile filter. *L. crescens* strain BT-1 was used in all experiments.

### Growth parameters assays for *L. crescens* cultures

Four variables were considered to study growth parameters of *L. crescens* including pH, aeration, temperature, and buffering capacity. These variables were evaluated using one-factor-at-a-time (OFAT) methodology [14] in the three growth media of *L. crescens* (Fig. 1)*.* pH optimization was the first parameter to be evaluated using an initial pH of 5.0 with increasing 0.2 values until salt precipitation occurs in the media. pH of all media and buffers was equilibrated at room temperature (24–25 °C). With the optimal growth pH, three different levels of aeration were further evaluated: 150, 200, and 250 rpm. Subsequently, optimal temperature for *L. crescens* growth was assessed at 22, 28 and 32 °C. Finally, four different buffers—monosodium phosphate, ACES, BIS-TRIS, and MES—with dissociation constants (pKa) close to the optimal growth pH of *L. crescens* were also evaluated at 10, 25, and 50 mM concentrations (Fig. 1).

#### Liberibacter crescens inoculum source

**Fig. 15:**
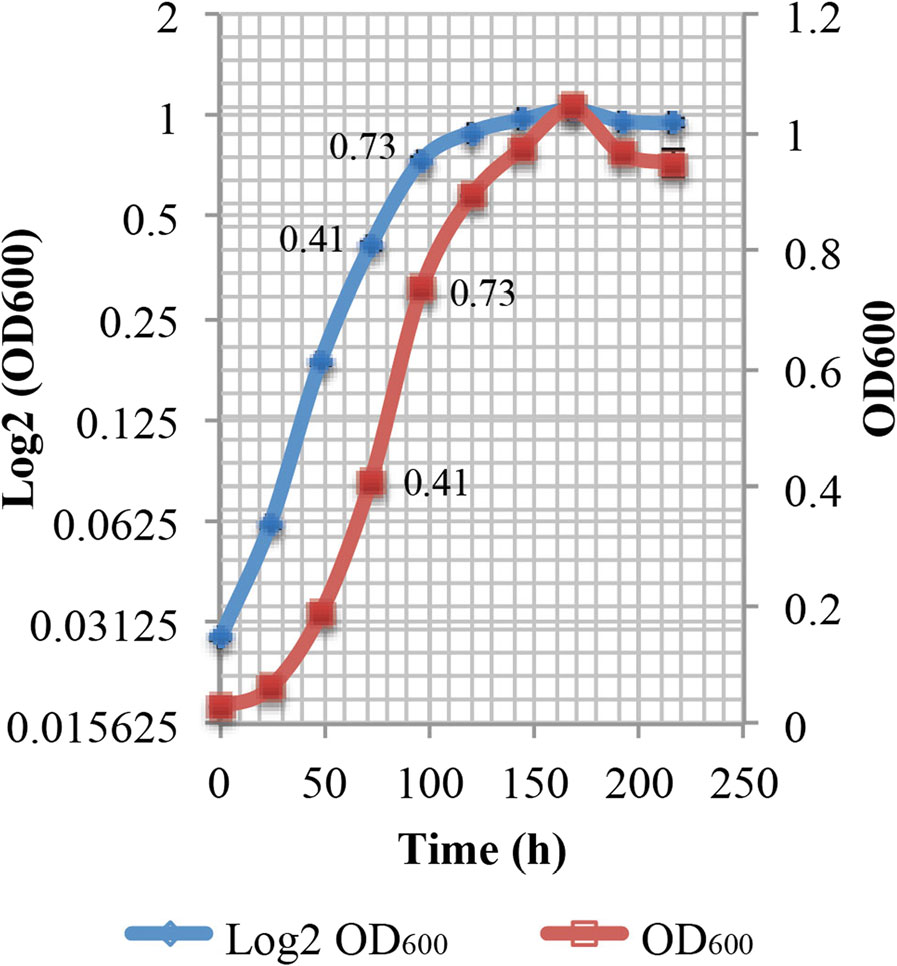
Growth curve of *Liberibacter crescens* inoculum source for optimal pH and aeration requirement experiments. 15 μL of glycerol stocks of *L. crescens* were activated in 2 mL of Hi-GI medium, subcultured in 5 mL of Hi-GI, and finally transferred to a 25 mL of Hi-GI in a 1:20 ratio inoculum: medium. Initial pH of Hi-GI was set to 5.92. Cultures were grown at 150 rpm and 28 °C. The inoculum was collected during middle to late exponential phase of growth at OD_600_~0.6–0.7 after 84 to 96 h prior to washing and inoculation in the different media

**Fig. 16:**
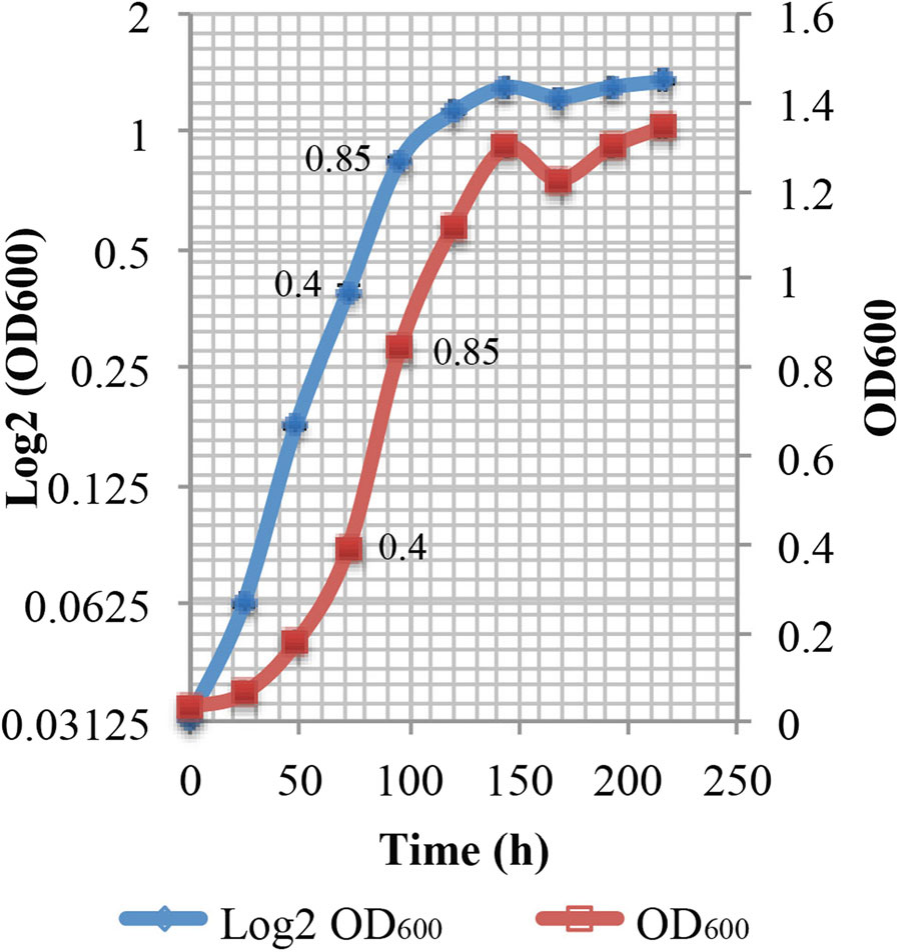
Growth curve of *Liberibacter crescens* inoculum source for temperature, buffer requirements and pH modulation experiments. 15 μL of glycerol stocks of *L. crescens* were activated in 2 mL of Hi-GI medium, subcultured in 5 mL of Hi-GI, and transferred to a 25 mL of Hi-GI in a 1:20 ratio inoculum: medium. Initial pH of Hi-GI was set to 5.92. Cultures were grown at 250 rpm and 28 °C. The inoculum was collected during middle to late exponential phase of growth at OD_600_~0.6–0.7 after 72 to 96 h prior to washing and inoculation in the different media

For the optimal pH and aeration requirement experiments, 15 μL of *L. crescens* glycerol stocks were activated and subcultured in Hi-GI pH 5.92 at 150 rpm, 28 °C to exponential phase (OD_600_~0.6–0.7, Fig. 15; see Additional file 1) prior to washing and inoculation in the different media. For optimal temperature and buffer requirements, *L. crescens* glycerol stocks were activated and subcultured in Hi-GI pH 5.92 at 28 °C, but using 250 rpm as the optimal aeration to an exponential phase (OD_600_~0.7–0.8, Fig. 16; see Additional file 1) prior to washing and inoculation. All inoculum cells were washed with a sterile buffer containing 7562.5 mg/L of ACES and 2836 mg/L of KOH, at pH 6.5 prior to inoculation at 1:20 ratio of inoculum: media in 25/30 mL of the different media. Growth and pH were monitored once a day for up to 13 days. Growth was examined through optical density (OD_600_) with the Synergy BioTek Multi-mode microplate reader (BioTek Instruments, Inc) using a 250 μL sample in a 96-well plate. pH was monitored using an AB15 pH meter (Fisher scientific) using 650 μL aliquots from each medium at room temperature (25–26 °C). All experiments were done by triplicate.

### pH modulation assays in *L. crescens* cultures

Three potential causes for the pH modulation in *L. crescens* cultures were evaluated in M15 chemically-defined medium: citrate metabolism, ornithine metabolism, and ammonium accumulation in the cell-free supernatant.

#### Citrate metabolism

*L. crescens* was grown in Hi-GI broth to exponential phase (OD_600_~0.7–0.8, Fig. 16; see Additional file 1), and washed with the previously described sterile ACES-KOH buffer pH 6.5 prior to inoculation in 30 mL of M15 and M15 without citrate. Optical density and pH were monitored as described previously once a day for 13 days.

#### Ornithine metabolism

*L. crescens* was grown in Hi-GI broth to exponential phase (OD_600_~0.7–0.8, Fig. 16; see Additional file 1), and washed with the previously described sterile ACES-KOH buffer pH 6.5 prior to inoculation in 30 mL of M15 and M15 without ornithine. Optical density and pH were monitored as described previously once a day for 13 days.

#### Determination of ammonium in M15 cell-free supernatant

*L. crescens* was grown in Hi-GI broth to exponential phase (OD_600_~0.7–0.8, Fig. 16; see Additional file 1), and washed with the previously described sterile ACES-KOH buffer pH 6.5 prior to inoculation in 30 mL of M15 media in triplicate. Optical density and pH were monitored once a day for a 13-day period. 50 μL of cell-free supernatant was also collected every day and stored at − 80 °C for subsequent ammonium analysis. Ammonium quantification over time was monitored using an ammonia/ammonium assay kit (Bioassay systems, Hayward, CA) following manufacturer recommendations. Briefly, cell-free supernatant samples and M15 negative control samples were diluted to 10-fold in ultrapure water. Standards were also prepared in 10-fold diluted M15 control in ultrapure water. The enzyme reaction contained 180 μL of assay buffer, 1 μL of enzyme, 8 μL of reconstituted NADH reagent, and 1 μL of ketoglutarate per sample. The blank control reaction contained the same components, except for the enzyme. Control and the experimental samples (20 μL) were mixed each with 180 μL of enzyme reaction and 180 μL of blank control reaction respectively in a 96 well plate. The plate was incubated for 30 min at room temperature. Absorbance was monitored at 340 nm using the Synergy BioTek Multi-mode microplate reader (BioTek Instruments, Inc). Ammonium concentrations were calculated accordingly using the manufacturer recommendations.

### Cell viability assay for *L. crescens* in optimal growth media conditions

Cell viability of *L. crescens* in the best optimal conditions was monitored using Presto Blue cell viability reagent (Invitrogen). *L. crescens* was first cultivated in Hi-GI broth to exponential phase (OD_600_~0.7–0.8, Fig. 16; see Additional file 1), and subsequently washed with the previously described sterile ACES-KOH buffer pH 6.5 prior to inoculation in 30 mL of M15, Hi-GI, or BM-7 media. 25 μL of Presto Blue were mixed with 225 μL of *L. crescens* culture samples in a black 96-well plate and incubated for two hours in the dark. Fluorescence was measured at 530/25 and 615/16 (excitation/emission) range with a gain of 45 using the Synergy BioTek Multi-mode microplate reader (BioTek Instruments, Inc). Cell viability was monitored once a day for 13 days along with optical density (OD_600_) of the cell cultures. Experiments were done by triplicate.

### Statistical analyses

Analysis of variance was used to determine relationships between the different media at any given condition. Post-hoc TukeyHSD test was subsequently used to determine statically significant traits in cell growth and pH among the media. Spearman rank (Rho) and Pearson linear correlations were used to correlate growth (OD_600_), pH, and ammonium concentrations over time. All tests were performed using R programming software version 3.3.2.

## Supplementary information

**Additional file 1.** Raw data for figures 2-16.

**Additional file 2.** Spearman and Pearson correlations between optical density and pH in L. crescens cultures with varying intial pH ranges.

**Additional file 3.** Spearman and Pearson correlations between optical density and pH in L. crescens cultures with varying concentrations of multiple buffers.

## Data Availability

All the data generated and analyzed during this study are included in this published article and its supplementary information files.

## Abbreviations

ACES: N-(2-Acetamido)-2-aminoethanesulfonic acid
AMTs: Ammonium transporters
ATR: Acid tolerance response
C: Carbon
C: Degree Celsius
*Ca*: *Candidatus*
CaCO_3_: Calcium carbonate
FBS: Fetal bovine serum
Fig: Figure
GDH: Glutamate dehydrogenase
GS/GOGAT: Synthetase/glutamate synthase
H^+^: Hydrogen ion
Hi-GI: Hi Grace’s insect medium
HLB: Huanglongbing
K^+^: Potassium ion
KOH: Potassium hydroxide
L: Liberibacter
MES: 2-(*N*-morpholino)ethanesulfonic acid
N: Nitrogen
NAD: Nicotinamide adenine dinucleotide
NaOH: Sodium hydroxide
NH4^+^: Ammonium ion
NO_3_^−^: Nitrate
OD_600_: Optical density
OFAT: One factor at a time methodology
*p*: *P* value
pH: Potential hydrogen
pKa: -log_10_ of the acid dissociation constant (K_a_)
PMF: Proton motive force
rpm: Revolutions per minute
TCA: Tricarboxylic acid
ZC: Zebra chip
