# Supplementary material for: Growth parameters of Liberibacter crescens suggest ammonium and phosphate as essential molecules in the Liberibacter-plant host interface
Source: BMC Microbiol. 2019 Oct 12;19:222. doi: 10.1186/s12866-019-1599-z (PMC6790036; doi:10.1186/s12866-019-1599-z)
Supplement: Supplementary file 2 — Additional file 2. Spearman and Pearson correlations between optical density and pH in L. crescens cultures with varying intial pH ranges. [file 12866_2019_1599_MOESM2_ESM.pdf]

Additional File 2. Spearman and Pearson correlations between optical density and pH in *L. crescens* cultures. Correlations were made over time for each medium.

**Media**	**Spearman Rho (R)**	**Spearman (pvalue)**	**Pearson (R)**	**Pearson (pvalue)**
**M15 pH 5.0**	0.602499199	2.4229E-05	0.696073264	3.08606E-07
**M15 pH 5.2**	0.923282786	3.23451E-18	0.885535563	6.76539E-15
**M15 pH 5.4**	0.986178111	7.51036E-33	0.94221438	1.33379E-20
**M15 pH 5.6**	0.990877421	1.92854E-36	0.898302564	7.17531E-16
**M15 pH 5.8**	0.98122403	3.28661E-30	0.923049767	3.42927E-18
**M15 pH 5.92**	0.937619068	5.90424E-20	0.945623357	4.08024E-21
**M15 pH 6.0**	0.95126711	4.80387E-22	0.902741463	3.06524E-16
**M15 pH 6.2**	0.962140434	3.40601E-24	0.846879151	1.56979E-12
**M15 pH 6.4**	0.970793764	2.05483E-26	0.866257384	1.26473E-13
**M15 pH 6.6**	0.922153869	4.28642E-18	0.918724812	9.82954E-18
**M15 pH 6.8**	0.755879347	7.14947E-09	0.917890364	1.19633E-17
**M15 pH 7.0**	0.773059161	1.9769E-09	0.869439671	8.0562E-14
**Hi-GI pH 5.0**	0.783135961	8.82056E-10	0.860329127	2.8437E-13
**Hi-GI pH 5.2**	0.954346444	1.33969E-22	0.969471508	4.92156E-26
**Hi-GI pH 5.4**	0.906540252	1.43221E-16	0.951644672	4.12621E-22
**Hi-GI pH 5.6**	0.933858512	1.83816E-19	0.956519386	5.15397E-23
**Hi-GI pH 5.8**	0.942932748	1.04551E-20	0.980459189	7.25305E-30
**Hi-GI pH 5.92**	0.94749237	2.06259E-21	0.984806762	4.91921E-32
**Hi-GI pH 6.0**	0.939318213	3.45273E-20	0.977183597	1.56177E-28
**Hi-GI pH 6.2**	0.953644046	1.80636E-22	0.978629106	4.27429E-29
**BM7 pH 5.0**	0.982365123	9.477E-31	0.882782448	1.06002E-14
**BM7 pH 5.2**	0.98780045	6.27483E-34	0.932577844	2.66543E-19
**BM7 pH 5.4**	0.987271482	1.45946E-33	0.984936671	4.14784E-32
**BM7 pH 5.6**	0.962272122	3.18059E-24	0.987056642	2.03576E-33
**BM7 pH 5.8**	0.934741201	1.41665E-19	0.970165953	3.12612E-26
**BM7 pH 5.92**	0.978037526	7.33986E-29	0.981245232	3.2138E-30
**BM7 pH 6.0**	0.944025562	7.17451E-21	0.968096205	1.17317E-25
**BM7 pH 6.2**	0.964603279	9.07477E-25	0.961810737	4.03869E-24
**BM7 pH 6.4**	0.931924554	3.21274E-19	0.958701074	1.87822E-23
**BM7 pH 6.5**	0.891063646	2.64986E-15	0.950450034	6.64861E-22
**BM7 pH 6.6**	0.964566661	9.26127E-25	0.962033583	3.59997E-24
**BM7 pH 6.8**	0.974137646	1.86179E-27	0.958649939	1.92437E-23
**BM7 pH 6.9**	0.966223419	3.60875E-25	0.961234888	5.41917E-24
**BM7 pH 7.0**	0.964455092	9.85215E-25	0.949014201	1.16165E-21
**BM7 pH 7.2**	0.954833005	1.08615E-22	0.953701032	1.76339E-22
**BM7 pH 7.4**	0.79694294	2.7168E-10	0.87405983	4.09703E-14
**BM7 pH 7.6**	0.429747261	0.004506851	0.653042808	2.76359E-06
**BM7 pH 7.8**	0.389911121	0.010693034	0.569638647	8.23795E-05
**BM7 pH 8.0**	0.202873283	0.197558344	0.331968883	0.031729579
**BM7 pH 8.2**	0.368293031	0.016403575	0.35727675	0.020190382
