# Supplementary material for: Growth parameters of Liberibacter crescens suggest ammonium and phosphate as essential molecules in the Liberibacter-plant host interface
Source: BMC Microbiol. 2019 Oct 12;19:222. doi: 10.1186/s12866-019-1599-z (PMC6790036; doi:10.1186/s12866-019-1599-z)
Supplement: Supplementary file 3 — Additional file 3. Spearman and Pearson correlations between optical density and pH in L. crescens cultures with varying concentrations of multiple buffers. [file 12866_2019_1599_MOESM3_ESM.pdf]

Additional file 3. Spearman and Pearson correlations between optical density and pH in *L. crescens* cultures. Correlations were made over time for each medium at different buffer concentrations. Hi = Hi-GI, Bis = Bis-Tris, NaP = Monosodium Phosphate

Media	Spearman Rho (R)	Spearman pvalue	Pearson (R)	Pearson pvalue
**M15**	0.747978944	4.74825E-06	0.932378692	5.35388E-13
**M15 no Bufer**	0.804751482	1.34021E-10	0.942229282	1.32711E-20
**M15 Bis 10mM**	0.557636946	0.000124789	0.797382113	2.61307E-10
**M15 Bis 25mM**	0.683640801	6.03752E-07	0.857373002	4.20136E-13
**M15 Bis 50mM**	0.673006741	1.04514E-06	0.892647081	2.00718E-15
**M15 Mes 10mM**	0.822064572	2.48302E-11	0.899615127	5.60289E-16
**M15 Mes 25mM**	0.657936374	2.19201E-06	0.880066151	1.63311E-14
**M15 Mes 50mM**	0.878180943	2.19102E-14	0.918197546	1.11314E-17
**M15 Aces 10mM**	0.785847643	7.04708E-10	0.90168266	3.76841E-16
**M15 Aces 25mM**	0.352967527	0.021859083	0.872546114	5.12783E-14
**M15 Aces 50mM**	0.538620867	0.000233459	0.80147415	1.80976E-10
**M15 NaP 10mM**	0.768600921	2.78883E-09	0.944310609	6.49526E-21
**M15 NaP 25mM**	0.454663961	0.00248677	0.777557858	1.38596E-09
**M15 NaP 50mM**	0.517427428	0.000449688	0.573257731	7.24521E-05
**Hi**	0.943527853	8.52465E-21	0.986634748	3.85178E-33
**Hi Bis 10mM**	0.862265243	2.19158E-13	0.942652076	1.1503E-20
**Hi Bis 25mM**	0.875478087	0.00000077	0.864572228	0.00000068
**Hi Bis 50mM**	0.898852843	6.47115E-16	0.97382085	2.36821E-27
**Hi Mes 10mM**	0.788652483	5.56788E-10	0.963488673	1.66984E-24
**Hi Mes 25mM**	0.846986397	1.54956E-12	0.979631246	1.65073E-29
**Hi Mes 50mM**	0.850971922	9.50127E-13	0.984441118	7.88877E-32
**Hi Aces 10mM**	0.759956451	5.32074E-09	0.980941898	4.41749E-30
**Hi Aces 25mM**	0.842971991	2.50139E-12	0.985344877	2.40329E-32
**Hi Aces 50mM**	0.818857315	3.43898E-11	0.977237977	1.48972E-28
**Hi NaP 10mM**	0.865691164	1.36875E-13	0.979051265	2.879E-29
**Hi NaP 25mM**	0.892062277	2.22514E-15	0.929454566	6.40357E-19
**Hi NaP 50mM**	0.646391089	3.7619E-06	0.928959351	7.33114E-19
**BM7**	0.593511488	3.4315E-05	0.956844076	4.44978E-23
**BM7 no bufer**	0.641162066	4.7693E-06	0.866458764	1.22957E-13
**BM7 Bis 10mM**	0.575965299	6.57503E-05	0.973456845	3.11109E-27
**BM7 Bis 25mM**	0.707505108	1.6154E-07	0.977434558	1.25472E-28
**BM7 Bis 50mM**	0.685613762	5.43944E-07	0.954258765	1.39098E-22
**BM7 Mes 10mM**	0.469962732	0.001687796	0.966365025	3.32223E-25
**BM7 Mes 25mM**	0.59053136	3.84247E-05	0.961058384	5.92495E-24
**BM7 Mes 50mM**	0.709307158	1.45463E-07	0.960630681	7.34265E-24
**BM7 Aces 10mM**	0.503934494	0.000667898	0.96073618	6.96575E-24
**BM7 Aces 25mM**	0.598904603	2.78826E-05	0.963928323	1.31585E-24
**BM7 Aces 50mM**	0.779056172	1.22911E-09	0.956828825	4.48071E-23
**BM7 NaP 10mM**	0.484793347	0.001139262	0.906291224	1.50694E-16
**BM7 NaP 25mM**	0.279381968	0.073160189	0.938981574	3.84464E-20
**BM7 NaP 50mM**	0.704075934	1.96783E-07	0.979593786	1.71193E-29
